# Effects of exercise on cognitive performance and ERP (P3, N2) in overweight and obese populations: a systematic review and meta-analysis

**DOI:** 10.1186/s13102-026-01593-0

**Published:** 2026-02-16

**Authors:** Chengcheng Zhu, Halijah Binti Ibrahim, Puyan Chi, Yilin Wang, Xiangyu Xu, Ting Peng, Aiping Chi

**Affiliations:** 1 Faculty of Educational Sciences and Technology, Universiti Teknologi Malaysia, Johor, 81310 Malaysia; 2 Shanghai Maritime University, Shanghai, 200240 China; 3 Xi’an Physical Education University, Xi’an, 710068 China; 4 Xi’an Siyuan University, Xi’an, 710038 China; 5 School of Sports, Shaanxi Normal University, Xi’an, 710100 China

**Keywords:** Exercise, Overweight, Obesity, Cognitive performance, ERP, P3 amplitude, N2 amplitude, Reaction time, Reaction accuracy

## Abstract

Individuals with overweight/obesity often show cognitive-control deficits, reflected in slower reaction times, lower accuracy, attenuated P3, and altered N2. We meta-analysed randomized controlled and within-subject crossover studies comparing exercise versus non-exercise controls during standard cognitive-control tasks. Primary outcomes were reaction time (RT), reaction accuracy (RA), P3 amplitude, and N2 amplitude; risk of bias was assessed with Cochrane RoB 2, and ERP reporting was evaluated with a checklist aligned to RoB-2 D4/D5.

Across 14 studies, exercise was associated with faster RT and larger P3 amplitude. RA and N2 showed directionally favorable but lower-certainty effects, reflecting substantial heterogeneity, small samples, and limitations in ERP quality reporting (e.g., sparse SNR/QC metrics; infrequent assessor blinding). Exploratory subgroup/meta-regression analyses offered limited explanatory power overall but suggested potential contributions of task paradigm, exercise mode, and design/comparator features—effects appeared larger in Stroop/Flanker than Go/No-Go and tended to be more pronounced for higher-arousal modes and under baseline/assessment-only controls and in designs incorporating baseline assessments—though these patterns should be interpreted cautiously given residual heterogeneity, sparse strata, and multiple comparisons.

Exercise thus confers measurable behavioral and electrophysiological gains in individuals with elevated BMI, consistent with cerebral blood-flow/neuroplasticity and catecholaminergic arousal mechanisms. Improved prespecification/multiplicity control and transparent ERP reporting are needed to strengthen future evidence and clarify dose–response and timing effects.

**Supplementary Information:**

The online version contains supplementary material available at 10.1186/s13102-026-01593-0.

## Introduction

### Overweight/Obesity and neurocognitive function

Overweight and obesity are now endemic across age groups and regions, and their consequences extend beyond cardiometabolic disease to the brain [1, 2]. Converging epidemiological, neuroimaging, and electrophysiological evidence links excess adiposity to alterations in executive functions—selective attention, inhibition, and conflict monitoring—and to atypical neural efficiency in fronto–cingulo–parietal networks [3–5].

Behaviorally, individuals with elevated BMI often show slower reaction time (RT) and, in some paradigms, reduced reaction accuracy (RA) on cognitive-control tasks (e.g., Go/No-Go, Flanker, Stroop) [3, 5]. At the neural level, event-related potentials (ERPs) provide temporally precise markers of these processes. In conflict tasks (e.g., Flanker/Stroop), N2 is commonly linked to conflict detection and the need for control, whereas P3 reflects the allocation of processing resources and context updating under increased task demands [6, 7]. In inhibition tasks (Go/No-Go), NoGo-related N2 and P3 are widely used indices of inhibitory engagement [6, 7]. Accordingly, the most theoretically informative behavioral indices often reflect control costs (e.g., incongruent–congruent or NoGo–Go contrasts) rather than overall task-average performance.

Studies in overweight/obese samples frequently report blunted P3 and attenuated (less negative) N2, patterns consistent with reduced allocation of cognitive resources and weaker control signals [3, 8, 9]. Importantly, these differences are observed even among otherwise healthy participants, suggesting that adiposity may alter neurocognitive function independent of overt disease [1, 2].

### Exercise and cognitive control

Exercise is a modifiable behavioral strategy that has been associated with improved cognitive function across the lifespan [10, 11]. Mechanistically, exercise is proposed to influence cognitive control via changes in cerebral perfusion and neuroplasticity-related signaling (e.g., BDNF), as well as arousal-related neurochemical pathways (e.g., catecholaminergic activity) [12, 13]. In the present review, these accounts are treated as plausible explanatory pathways; detailed mechanistic integration is provided in the Discussion.

Two complementary bodies of evidence motivate the present synthesis. First, randomized and quasi-experimental studies in general-weight or mixed samples indicate that exercise can yield small-to-moderate improvements in performance on inhibition/interference tasks such as Go/No-Go, Flanker, and Stroop [14–16]. Second, EEG/ERP-focused syntheses suggest that exercise can alter neural indices of cognitive processing. For example, Hosang [17] summarized exercise-related effects on EEG-recorded neural oscillations, and Kao [18] reported meta-analytic evidence for acute exercise effects on P3 amplitude and latency.

However, these broader syntheses typically pool heterogeneous populations and task metrics, and they rarely quantify behavioral and ERP outcomes jointly within overweight/obese samples [19–21]. As a result, it remains unclear whether exercise-related changes in behavioral control costs and ERP markers co-vary in individuals with elevated BMI, and whether the magnitude and moderators of effects differ from patterns observed in normal-weight samples [12, 18, 21].

### Gaps at the behavior–ERP interface in elevated BMI

Despite growing primary research, three gaps limit cumulative inference. First, prior syntheses often mix normal-weight and overweight/obese samples or focus exclusively on either behavioral or ERP outcomes. This limits the ability to determine whether exercise simultaneously benefits behavioral indices of executive control (RT/RA and control-cost contrasts) and ERP markers (P3/N2) in populations with elevated BMI [12, 22].

Second, the field lacks a risk-of-bias–aware, ERP-literate appraisal. ERP outcomes are sensitive to methodological choices such as referencing and filtering, artifact handling/ICA, trial counts and retention, prespecified windows/regions of interest, and multiplicity control [23–25]. Without structured evaluation of these features, pooled effects may be misestimated [26].

Third, the population-specific question remains under-specified: overweight/obesity may constitute a distinct neurocognitive context in which effect sizes, temporal dynamics, and moderators differ. Elevated BMI is associated with metabolic and inflammatory alterations and may be accompanied by reduced cardiorespiratory fitness, factors that can plausibly influence executive-control efficiency and neuroelectric indices of resource allocation and control engagement. Consequently, moderators such as exercise intensity/dose, task family (conflict vs. inhibition), and intervention type (acute vs. training) may operate differently in overweight/obese samples than in general-weight populations. Yet moderator evidence in this population is limited and inconsistent, and earlier syntheses have largely focused on P3 alone with mixed conclusions [18, 19, 21]. Addressing these uncertainties requires integrating behavioral and electrophysiological outcomes within a unified quantitative framework while systematically testing candidate moderators and heterogeneity.

### Objectives and an ERP-literate meta-analytic approach

To elucidate the cognitive and neural effects of exercise in individuals with elevated BMI, we conducted a meta-analysis of randomized controlled trials and within-subject crossover studies comparing exercise with non-exercise control during standard cognitive-control tasks (Go/No-Go, Flanker, Stroop, or comparable paradigms). We focused on four primary outcomes—reaction time (RT), reaction accuracy (RA), P3 amplitude, and N2 amplitude—to integrate behavioral performance with electrophysiological markers. Consistent with executive-control theory and task conventions, we prioritized theoretically informative indices where available (e.g., incongruent–congruent and NoGo–Go contrasts) to align behavioral synthesis with the functional interpretation of N2/P3 across task families.

Given variability in exercise prescription and EEG methodology, we extracted intervention characteristics and control activities, and evaluated these—together with BMI category and demographics—as candidate moderators in subgroup and meta-regression analyses [18, 27]. We assessed risk of bias using Cochrane RoB 2 (including the version for crossover trials) and summarized the adequacy of ERP reporting using a structured checklist [25, 26]. By integrating behavioral and ERP outcomes within a single framework and explicitly addressing heterogeneity in study design and reporting, this review provides a coherent evaluation of exercise-related cognitive effects in overweight/obese samples and offers methodological guidance for future trials.

## Methods

### Eligibility criteria

This review followed a prospectively registered protocol (PROSPERO: CRD420251005357) and adhered to the PRISMA 2020 reporting guideline [28]. Eligibility was prespecified using the PICOS framework [29]. We included peer-reviewed trials published in English that examined the effects of exercise—either acute (single session) or long-term (multi-week training)—on individuals with overweight or obesity, and reported ≥ 1 of the following: reaction time (RT), reaction accuracy (RA), P3 or N2 amplitude.

#### Participants

Adults (≥ 18y): WHO/International definitions—overweight BMI ≥ 25.0 kg/m², obesity BMI ≥ 30.0 kg/m² [30]. East-Asian alternatives were accepted when used by study authors: China/WGOC ≥ 24.0/≥ 28.0 [31], Taiwan MoHW 24.0–26.9/≥ 27.0 [32].

Children & adolescents (< 18 y): accepted references were WHO 2007 BMI-for-age ( > + 1SD/> +2SD) [33], CDC 2000 percentiles (≥ 85th/≥ 95th) [34], or IOTF/Cole cut-points [35]. 

#### Interventions and comparators

Interventions: aerobic, resistance, or combined exercise, delivered as acute or multi-week training, supervised or not, in laboratory, clinical, or community settings (American College of Sports Medicine) [36]. We extracted mode, session duration/frequency, intensity (e.g., %HRmax, %VO₂ max/HRR, RPE), and the interval between exercise and ERP assessment [29].

Comparators: non-exercise controls (seated rest, quiet reading/usual activity) or time/attention-matched sedentary activities (e.g., light stretching, simple cognitive tasks) without an effective exercise component. 

#### Outcomes

The primary outcomes were reaction time (RT, ms) and reaction accuracy (RA, % correct) during cognitive task paradigms, as well as P3/P300/P3b amplitude (µV) and N2 amplitude (µV) assessed via event-related potentials (ERPs). Studies were eligible for inclusion if they reported at least one of these outcomes. 

#### Study designs

Eligible designs were randomized controlled trials (parallel-group or within-subject crossover) comparing an exercise arm with a non-exercise control, using pre–post or posttest-only outcome assessment [37]; crossover trials required adequate control of carryover/washout or were flagged in risk-of-bias [29]. Non-randomized designs, single-arm studies, case reports, animal studies, and conference abstracts without sufficient data were excluded.

### Information sources, search strategy, and study selection

We searched four bibliographic databases: CLIB – The Cochrane Library, Embase.com, PubMed, and Web of Science. No restrictions were placed on the search start date; searches covered records up to 30 August 2025. The review included studies published in English only. Additional records were identified by backward citation checking and by contacting authors/experts when necessary.

Search Strategies combined controlled vocabulary and free-text terms for exercise/physical activity and event-related potentials. The PubMed strategy (adapted for other databases) was: ((((((exercise[MeSH Terms]) OR (exercise*[Title/Abstract])) OR (aerobic exercise[MeSH Terms])) OR (exercise, physical[MeSH Terms])) OR (physical exercise[MeSH Terms]) OR (physical activity[MeSH Terms]) OR (training, Exercise[MeSH Terms])) AND (((((((((event-related potentials[MeSH Terms]) OR (event-related potential*[Title/Abstract])) OR (Event Related Potential[MeSH Terms])) OR (Event Related Potential*[Title/Abstract])) OR (Evoked Potential*[MeSH Terms])) OR (Evoked Potential*[Title/Abstract])) OR (ERP[Title/Abstract])) OR (ERPs[Title/Abstract])))) NOT ((animals[MeSH Terms]) NOT (humans[MeSH Terms]))

Full database-specific syntaxes (including Embase Emtree terms, Cochrane Library syntax, and Web of Science topic fields) will be provided in the *Supplement 1*; terms were iteratively refined by an experienced information specialist.

All records were exported to EndNote X9 (v19.0.0) for management and deduplication, then imported into the screening platform. Two reviewers (ZCC, CPY) independently screened titles/abstracts and full texts against the prespecified eligibility criteria; discrepancies were resolved by consultation with a third reviewer (CAP). Reasons for full-text exclusion were recorded. The selection process will be presented in a PRISMA 2020 flow diagram [28].

### Data extraction and coding

Three reviewers (ZCC, CPY, PT) independently extracted data for behavioral and ERP outcomes. The following descriptors were recorded for each study: (1) first author and publication year; (2) sample characteristics (group sizes, age, sex, BMI definition/category); (3) study design (parallel RCT or within-subject crossover); (4) cognitive task family (conflict: Flanker/Stroop; inhibition: Go/No-Go; other executive-control paradigms); (5) exercise intervention details (mode, session duration, frequency, intensity, and supervision); (6) exercise-to-assessment interval for acute studies; (7) control activity; and (8) outcome reporting features relevant to ERP acquisition and analysis. Extracted datasets were cross-checked jointly, and discrepancies were resolved through discussion until consensus.

From each eligible comparison we extracted summary statistics as reported in the original analyses. Depending on availability, we extracted either posttest means/SDs or change-from-baseline values (and their variability). For within-subject/crossover trials, paired contrasts were treated as within-participant estimates [29, 38]; when the within-person correlation was not reported, we assumed *r* = 0.5 in the primary analysis and conducted sensitivity analyses using *r* = 0.3 and *r* = 0.7. When only medians and IQRs were reported, values were cautiously converted using recommended formulae [39, 40]. If only error rates were provided, RA was computed as 1 − error rate.

Behavioral and ERP outcomes. To align behavioral indices with executive-control theory and the functional interpretation of N2/P3 across task families, we prioritized control-cost contrasts whenever available. For conflict tasks (e.g., Flanker/Stroop), we extracted incongruent–congruent (Incong–Cong) differences for RT/RA and, correspondingly, task-relevant P3/N2 contrasts (incongruent vs. congruent or difference-wave indices when reported/derivable). For inhibition tasks (Go/No-Go), we extracted NoGo–Go differences for RT/RA and, correspondingly, NoGo-related P3/N2 effects (NoGo–Go contrasts when available). If studies reported only condition-specific values (e.g., separate means/SDs for incongruent and congruent, or NoGo and Go), we computed the corresponding contrasts using standard formulas (see § 2.5.1).

For ERP outcomes, we extracted P3 and N2 amplitudes (µV) and their variance from prespecified midline regions consistent with typical component topographies (P3: centro-parietal; N2: fronto-central), and recorded the a priori time windows and ROIs as reported by study authors. When multiple electrodes within the prespecified ROI were provided, we extracted the ROI summary if available; otherwise, we prioritized the canonical midline electrode within the ROI (e.g., Pz/CPz for P3; FCz/Cz for N2) consistent with the study’s prespecified analysis. When both peak and mean amplitudes were reported, we prioritized mean amplitude within the prespecified window to improve comparability across studies.

For the small minority of studies (*n* = 2) in which contrast-based indices could not be derived from the reported data, we extracted task-relevant single-condition values (NoGo for Go/No-Go tasks; incongruent for conflict tasks) and flagged these effects as “single-condition” for sensitivity analyses.

ERP methodological descriptors (for reporting adequacy and RoB support). We extracted ERP methodological parameters including reference scheme (e.g., average mastoids/average reference), band-pass filters, sampling rate, artifact handling (ICA and/or rejection thresholds), trial counts and retention, prespecified windows/ROIs, and multiplicity control (e.g., predefined windows, FDR/cluster-based procedures) [23, 25, 41]. These parameters were used to appraise ERP reporting adequacy and to inform RoB-2 judgments and sensitivity analyses; they were not synthesized as outcomes.

Exercise was coded at the session/program level: intervention type (acute, training), mode (aerobic, resistance, combined), session duration (min), and intensity (preferentially %HR_max or %VO₂max/HRR; otherwise RPE or workload) consistent with exercise prescription guidelines (ACSM) [36]. For acute studies, we recorded the timing before ERP assessment and binned it a priori (≤ 15, 15–30, ≥ 31 min) while retaining exact minutes for meta-regression [18, 42]; for training studies, we extracted the post-intervention assessment closest to program end (and baseline values when change scores were analyzed). When trials included multiple eligible exercise arms, we included each arm to avoid selective inclusion bias and to enable exploration of dose/intensity effects, using standard approaches to maintain statistical independence [29].

Control conditions were coded into three a priori categories and used as moderators: (i) Baseline/assessment-only (no added activity beyond assessments/preparation), (ii) Passive cognitive engagement (low-demand sedentary activities such as quiet reading or viewing/listening tasks, without meaningful metabolic or cognitive training load), and (iii) Cognitive engagement (sedentary activities with higher cognitive demands designed to match attention/motivation, e.g., computerized tasks or challenging puzzles without exercise components), consistent with prior exercise-cognition taxonomies distinguishing resting/attention-matched comparators [18, 43]. For crossover trials, the non-exercise period served as the comparator provided washout/carryover control was adequate [38].

Handling of unreported or inapplicable items. Items not reported by the original studies were coded as NI (No Information) and, where relevant, explored in sensitivity analyses using missingness indicators; items not applicable to a given design (e.g., Exercise timing before ERP in multi-week training trials) were coded as N/A and excluded from moderator analyses for that construct. All data extraction was performed in duplicate, with discrepancies resolved by consensus. Analyses including NI indicators are exploratory and should be interpreted as proxies for reporting adequacy rather than causal moderators.

### Risk of bias assessment

Risk of bias (RoB) was assessed with the Cochrane RoB-2 tool [26], using the parallel-group and crossover variants as appropriate. Two reviewers (ZCC, CPY) judged each study independently, with disagreements resolved by a third reviewer (CAP). Judgements used the RoB-2 traffic-light scale (Low, Some concerns, High) at the domain level and then overall. Domains were operationalised for exercise-ERP trials as follows:

- D1: Randomization process (random allocation, allocation concealment, and baseline comparability);
- D2: Deviations from intended interventions (between-group/within-subject analysis appropriate, exercise intervention sufficiently evaluated/reported);
- D3: Missing outcome data (missingness acceptable, handling appropriate/balanced);
- D4: Measurement of the outcome (assessor blinding; ERP acquisition/pre-processing adequate);
- D5: Selection of the reported result (point estimates with variability adequately reported, evidence of prespecification/multiplicity control sufficient).

Item-level codes were Y/PY/PN/N/NI (Yes/Probably yes/Probably no/No/No information). Overall RoB was rated Low only if all domains were Low; High if any domain was High or if multiple domains had Some concerns; otherwise Some concerns [26]. Where key information was not reported (NI), this contributed to Some concerns unless the direction of bias could be ruled out. This conservative rule was adopted because missing methodological and analytic details—particularly for ERP acquisition/preprocessing choices, prespecified windows/ROIs, and multiplicity control—can plausibly influence effect estimation and cannot be assumed to be neutral in direction or magnitude.

Because ERP outcomes are method-sensitive, we used a structured ERP reporting checklist to inform RoB-2 domains D4 (measurement) and D5 (selection of the reported result) and to predefine sensitivity analyses. The checklist captured: (i) referencing and filtering settings, artifact-handling procedures (e.g., ICA, rejection thresholds), and sampling rate—mapped to D4-2 (ERP acquisition/pre-processing adequate? ); (ii) trial counts/retention as a proxy for signal-to-noise adequacy—mapped to D4-4; (iii) a priori time windows and ROIs (P3: centro-parietal; N2: fronto-central)—mapped to D4-3 (ERP analysis/reporting adequate? ); (iv) multiple-comparison control (predefined windows, FDR/cluster procedures)—mapped to D5-2 (prespecification/multiplicity control sufficient); and (v) analyst blinding/automation—mapped to D4-1 (assessor blinding). These items align with contemporary ERP/MEEG reporting recommendations [23, 25, 41]. Note that D5-1 (point estimates with variability) is not part of the checklist items and was checked separately (i.e., whether means with SD/SE or 95% CIs were reported to permit effect-size computation). Traffic-light outputs (domain-level and overall) are presented in the RoB table.

### Effect measures and data synthesis

#### Primary meta-analysis

We synthesized effects using random-effects models to accommodate between-study heterogeneity. Outcomes were coded so that benefit favors exercise (reduced RT control costs; higher RA; larger P3 contrast amplitudes; and more negative N2 contrast amplitudes). To harmonize inference across outcomes and trial designs (parallel and within-subject), we used Hedges’ g (small-sample corrected standardized mean difference) as the common effect size [44].

For parallel-group RCTs, Hedges’ g was computed for the exercise–control contrast using change scores when available (preferred) or posttest values when baseline comparability was adequate (per RoB-2 D1) [29]. The standardized effect was calculated as the mean difference divided by the pooled SD and multiplied by Hedges’ small-sample correction factor; when only adjusted contrasts (e.g., ANCOVA) were reported, we used them when sufficient information was provided to derive standard errors, otherwise we used unadjusted contrasts from descriptive statistics [29].

For within-subject/crossover trials, we prioritized task-relevant within-participant contrasts (e.g., Incong–Cong; NoGo–Go) for both behavioral and ERP outcomes. When only condition-specific means/SDs were available, the SD of the paired difference was derived as

$$\:SD\varDelta\:=\sqrt{S{D}_{1}^{2}+S{D}_{2}^{2}-2r\cdot\:S{D}_{1}\cdot\:S{D}_{2}}$$

and the mean difference was standardized by SDΔ to obtain paired Hedges’ g [29]. Because within-person correlations (r) were seldom reported, we used *r* = 0.5 in the primary analysis and conducted prespecified sensitivity analyses using *r* = 0.3 and *r* = 0.7 [45].

When a single study contributed multiple eligible exercise arms, we maintained statistical independence using standard approaches (e.g., splitting shared control groups or combining arms as appropriate), consistent with Cochrane guidance [29, 38]. For acute studies with multiple post-exercise time points, we followed the prespecified hierarchy described in § 2.3 for primary extraction. All statistical analyses were conducted in Stata/SE 11.0 [46].

#### Heterogeneity

We appraised heterogeneity by visual inspection of forest plots (overlap of 95% CIs) and by statistics. Primary pooled effects were estimated using inverse-variance random-effects models with the DerSimonian–Laird estimator as implemented in Stata (metan, random); heterogeneity was summarized with I² (95% CI) and assessed with Cochran’s Q (two-sided, α = 0.10 for the omnibus test of a common effect). Following convention, approximately 25%/50%/75% were interpreted as low/moderate/high heterogeneity [47]. Where applicable, subgroup-specific I² values and 95% prediction intervals are reported alongside within-subgroup pooled effects and between-subgroup tests.

#### Subgroup analyses

We ran prespecified random-effects subgroup analyses for BMI, gender, age, experimental design, task paradigm, intervention type, exercise mode, exercise intensity, exercise duration, timing before ERP assessment, and control activity. For each factor we report:

(i) between-subgroup difference using a Knapp–Hartung F-test with df₁ = k levels − 1 and df₂ = total studies − k (shown as F(df₁, df₂), p), and the factor-level I²; and.
(ii) within-level pooled effects as Hedges’ g [95% CI], along with level-specific I². Levels with k = 1 or NI (no information) are listed but not included in the between-group test. All subgroup analyses were treated as exploratory and interpreted alongside meta-regression.

#### Meta-regression

We ran random-effects meta-regressions (REML with Knapp–Hartung inference) to explore residual heterogeneity [48, 49]. Moderators matched those in § 2.5.3. Categorical moderators were entered as sets of indicator variables with prespecified reference levels (reference coding was statistical rather than implying a theoretical benchmark). We report the regression coefficient b (SE), t, p, and, for each categorical factor, the omnibus between-level test F (df₁, df₂), p. Residual heterogeneity indices (e.g., and I²_res) are reported where available. Meta-regression findings were interpreted cautiously given small and unbalanced numbers of studies across moderator levels.

#### Sensitivity and Small-study effects

To evaluate robustness, we performed leave-one-out analyses [50] for each primary outcome (RT, RA, P3, N2). Because variance estimation for within-subject contrasts depends on the assumed within-person correlation, we also conducted prespecified sensitivity analyses by repeating each meta-analysis under *r* = 0.3, *r* = 0.5 (primary), and *r* = 0.7. Potential small-study and reporting biases were examined with funnel plots and Egger’s regression (α = 0.05) when k ≥ 10; when k < 10, interpretation relied on visual inspection and narrative appraisal due to low power [51]. Where asymmetry was suspected, we applied Duval & Tweedie’s trim-and-fill to estimate the potential impact of missing studies on pooled effects [52].

### Certainty of evidence

Certainty of evidence. We appraised certainty using GRADE [53, 54] for each primary outcome (RT, RA, P3, N2), starting at High (all RCTs) and rating down for risk of bias (RoB-2), inconsistency (I²/overlap), indirectness, imprecision (95% CI/OIS), and publication bias (funnel/Egger). A SoF (Summary-of-Findings) table presents pooled effects (Hedges’ g), 95% CIs, number of studies/participants, final GRADE ratings, and footnoted reasons.

## Results

### Study selection

**Fig. 1:**
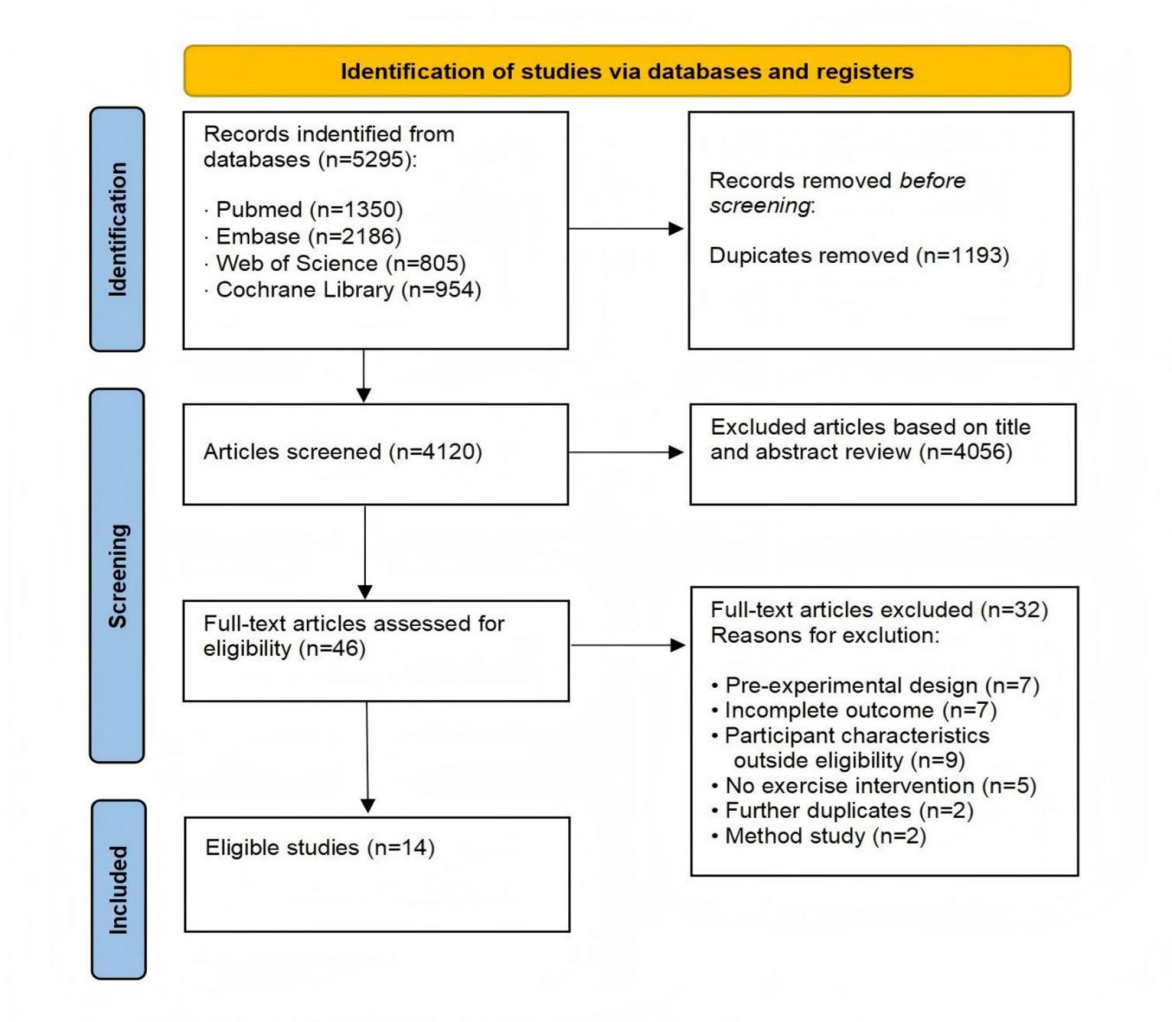
PRISMA flow diagram of systematic search and study selection

The search identified 5,295 records across four databases (PubMed, *n* = 1,350; Embase, *n* = 2,186; Web of Science, *n* = 805; Cochrane Library, *n* = 954). After removal of 1,193 duplicates, 4,102 unique records remained for title/abstract screening. Of these, 4,056 records were excluded as clearly irrelevant to the review question. We retrieved 46 full-text articles for eligibility assessment; 32 were subsequently excluded for the following reasons: pre-experimental design (*n* = 7), incomplete outcome data (*n* = 7), participant characteristics outside eligibility (*n* = 9), no exercise intervention (*n* = 5), duplicate at full text (*n* = 2), and methodological study (*n* = 2). Ultimately, 14 studies met all inclusion criteria and were included in the quantitative synthesis (Fig. 1. PRISMA flow diagram).

### Characteristics of included studies

#### Sample and general features

As summarized in Table 1, fourteen trials were included, enrolling individuals with overweight (*n* = 6) or obesity (*n* = 8), with a total of 880 participants. Most studies recruited young adults (*n* = 13), with one trial conducted in children/adolescents. Sex composition varied across studies (male-only, female-only, and mixed-sex samples), and BMI status was defined using recognized adult criteria (WHO or East-Asian alternatives) or age-adjusted child/adolescent references, consistent with the registered protocol (Table 1).

**Table 1:** Summary of characteristics of included studies

Summary of characteristics of included studies									
Trials	*N*	Age(M ± SD)	Gender (F%)	BMI (M ± SD)	Main outcome measures	Task Paradigm	Exercise timing before ERP	Experimental design	
								Exercise Characteristics	Comparison
Bailey et al. (2021) [55]	210	22.5 ± 3.7	NI (Both male and female)	25.4 ± 3.1	RT, RA, N2: 200-300ms P3༚400-550ms	Go/nogo task Flanker task	20–25 min(go/nogo) 25–30 min(flanker)	Design: XO (within), 3-session, post-only Sessions: slow jogging 35% VO₂max; jogging 70% VO₂max, CON Mode: aerobic Duration: 40 min Intensity: low (35% VO₂max) / high (70% VO₂max)	Comparator: seated rest performing routine seated tasks or watching a low-arousal/neutral video (CON).
Carbine et al. (2024) [56]	138	25.67 ± 8.80	54.35%	26.54 ± 7.32	N2: 200-300ms P3༚400-550ms	Go/nogo task	15 min	Design: XO (within), 2-session, post-only Sessions: treadmill walking, CON Mode: aerobic Duration: 45 min Intensity: moderate (MET 4.3–5)	Comparator: seated academic/health reading (CON).
Hanlon. (2011) [57]	35	32.4 ± 9.1	100%	28.3 ± 6.6	LPP: 400-550ms P300༚300-500ms	Passive viewing of images	45–60 min	Design: XO (within), 2-session, post-only Sessions: treadmill walking, CON Mode: aerobic Duration: 45 min Intensity: moderate (MET 4.3–5)	Comparator: body-composition testing only, no exercise (CON).
Kuang et al. (2025) [58]	19	29.9 ± 7.5	63% F	30.0 ± 3.64	RT, RA, P3b: 300-500ms	Flanker task	NI	Design: XO (within), 2-session, pre–post Sessions: SOC + SIT (seated social interaction); MPA + SIT (moderate-intensity physical activity + sitting) Mode: aerobic Duration: 17.5 min Intensity: moderate (55% HRR)	Comparator: SOC + SIT (quiet social interaction, seated reading)
Logan et al. (2021) [59]	206	8.72 ± 0.63	56.31%	33.83 ± 8.12 (Whole Body Fat %)	RT, RA, P3: 300-600ms	Flanker task	N/A	Design: parallel RCT, pre–post Groups: normal-weight (PA/CON) and obesity (PA/CON) strata Mode: combined (dance + skills) Duration: 120 min/day, 150 days Intensity: moderate	Comparator: usual daily activity (OB/CON)
Lv et al. (2024) [60]	59	20.36 ± 1.78	0%	24.91 ± 1.82	RT, RA, N2: 200-300ms P3༚300-500ms ERN:0-100ms	Stop signal task	NI	Design: XO (within), 4-session, pre–post Sessions: MICT (moderate-intensity continuous training), BWT (body-weight training), MBE (mind-body exercise), CON Modes: aerobic / resistance / mind–body Durations: 30 min (MICT), 20 min (BWT), 20 min (MBE) Intensity: moderate (MICT, 60–70% HRmax); high (BWT, e.g., Tabata); low (MBE, e.g., Tai Chi)	Comparator: no exercise, maintain usual activity (CON)
Sun and Ren. (2024) [61]	28	21.29 ± 2.67	50%	25.20 ± 3.53	RT, RA, P3: 400-540ms	Flanker task	≤ 10 min	Design: XO (within), 3-session, post-only Sessions: CMIAE (continuous moderate-intensity aerobic exercise), HIIE (high-intensity intermittent exercise), CON Mode: aerobic / HIIE Duration: 30 min (CMIAE), 16 min (HIIE) Intensity: moderate (65–70% HRmax, CMIAE); high (50–90% HRmax, HIIE)	Comparator: quiet seated rest (CON)
Tsai et al. (2019) [52] 66]	54	23.68 ± 2.39	0%	26.71 ± 4.73	RT, RA, N2: 120-250ms P3༚250-400ms	Visuospatial attention task	N/A	Design: parallel RCT, post-only Groups: healthy weight (HW); obesity with regular exercise (ORE); obesity with sedentary lifestyle (OSL) Mode: combined Duration: ≥ 30 min per session, ≥ 3 sessions/week within 24 months Intensity: moderate (METs ≈ 3.6)	Comparator: OSL (maintain usual sedentary lifestyle)
Wang et al. (2024) [62]	18	24.50 ± 5.13	0%	34.60 ± 4.21	RT, RA, N2: 200-300ms P3༚350-500ms	Flanker task	≤ 15 min	Design: XO (within), 3-session, post-only Sessions: LIE (low-intensity), MIE (moderate-intensity), CON Mode: aerobic (cycle ergometer) Duration: 30 min Intensity: low (40–50% HRmax), moderate (65–70% HRmax)	Comparator: time-matched seated rest (CON)
Wen et al. (2022) [54] [67]	32	33.90 ± 6.44	100%	29.50 ± 3.89	RT, RA, P2: 170-270ms N2: 200-400ms P3༚300-600ms	Stroop task	N/A	Design: parallel RCT, pre–post Groups: exercise group (EG), control group (CG) Mode: combined (aerobic dance + dumbbell resistance) Duration: 30 min×5/week, 12 weeks Intensity: moderate (55–59% HRR)	Comparator: usual lifestyle, no structured exercise (CG)
Wen and Tsai. (2020) [53] [68]	32	33.03 ± 6.63	100%	30.95 ± 4.92	RT, RA, P2: 150-275ms N2: 200-400ms P3༚300-800ms	Stroop task	28.4 min	Design: parallel RCT, pre–post Groups: exercise group (EG), control group (CG) Mode: combined (aerobic dance + dumbbell resistance) Duration: 30 min Intensity: moderate (55% HRR)	Comparator: 30-min seated rest (CG)
Xie et al. (2020) [63]	16	24.50 ± 5.09	0%	34.34 ± 4.39	RT, RA, P3: 300-400ms LPP༚600-1100ms	Flanker task	≤ 15 min	Design: XO (within), 2-session, post-only Sessions: HIIE (high-intensity intermittent exercise), CON Mode: HIIE Duration: 30 min Intensity: high (80–90% HRmax)	Comparator: time-matched seated rest (CON)
Xie et al. (2024) [64]	15	24.60 ± 5.29	0%	33.88 ± 4.22	RT, RA, P3: 400-600ms LPP༚700-1100ms	Flanker task	≤ 15 min	Design: XO (within), 2-session, post-only Sessions: HIIE (high-intensity intermittent exercise), CON Mode: HIIE Duration: 30 min Intensity: high (80–90% HRmax)	Comparator: time-matched seated reading (CON)
Xie et al. (2025) [65]	18	24.50 ± 5.13	0%	34.60 ± 4.21	RT, RA, N2: 200-300ms P3༚350-600ms	Go/nogo task	≤ 15 min	Design: XO (within), 3-session, post-only Sessions: LIE (low-intensity), MIE (moderate-intensity), CON Mode: aerobic (cycle ergometer) Duration: 30 min Intensity: low (40–50% HRmax); moderate (65–70% HRmax)	Comparator: time-matched seated rest (CON)

*Abbreviations*: *NI* No information, *N/A* Not applicable, *CON* Control (group), *XO* (*within*) Within-subject randomized crossover, *Parallel RCT* Parallel-group randomized controlled trial, *pre–post* Baseline and post-intervention assessments, *post-only* Posttest assessment only.

#### Task class, outcomes, and key ERP features

Studies employed standard executive-control paradigms spanning inhibition and conflict task classes, primarily Go/No-Go (*n* = 3), Flanker (*n* = 7), and Stroop (*n* = 2). All trials reported at least one behavioral outcome (RT and/or RA) and at least one ERP outcome (P3 and/or N2). P3 was typically quantified over centro-parietal regions and N2 over fronto-central regions, although EEG acquisition and preprocessing decisions (e.g., reference scheme, filtering, artifact handling, trial retention) varied in completeness and were documented in the ERP reporting checklist (*Supplement Table *S1). Additional ERP components were occasionally reported (e.g., LPP [57, 63, 64], P2 [67, 68], and ERN [60]) but were not meta-analyzed due to insufficient extractable contrasts.

#### Study design and intervention characteristics relevant to heterogeneity

Most studies used randomized within-subject crossover designs (*n* = 10), whereas three trials were parallel-group RCTs [59, 67, 68]. Interventions comprised both acute exercise and multi-week training, with acute protocols predominantly aerobic or high-intensity interval formats, and training protocols typically combined aerobic and resistance components. Control conditions were operationalized using our a priori taxonomy and were most often seated rest/baseline (assessment-only), with a smaller number using passive cognitive engagement [55, 56] or cognitively engaging sedentary comparators matched for time/attention [58, 64] (see Table 1). Together, variation in task class (inhibition vs. conflict), intervention type (acute vs. training), and comparator activity constituted the main sources of clinical and methodological heterogeneity evaluated in subgroup and meta-regression analyses.

### Risk of bias within studies

In acute studies (*n* = 11), ERPs were recorded immediately to ≥ 30 min after exercise; based on prior work [18, 42], we coded three a priori bins (≤ 15 min, 15–30 min, ≥ 31 min) and retained exact minutes for meta-regression. In training studies (*n* = 3), ERPs were assessed post-program (and at baseline when change scores were available).

Using the Cochrane RoB 2 tool (Table 2), most trials were judged to have some concerns overall (*n* = 13), and only one study was rated high risk of bias [66]. The Some concerns ratings were mainly driven by unclear allocation concealment (D1), unreported assessor blinding (D4), incomplete prespecification/multiplicity control (D5), and occasional “NI” judgements for trial retention—issues that largely reflect reporting limitations rather than unequivocal implementation failures. In particular, for domains D1-2 (allocation concealment) and D4-1 (assessor blinding), it is inherently difficult to achieve and transparently report rigorous concealment and blinding procedures in exercise-based intervention trials, especially when interventions are delivered face-to-face or in groups [29], and these features contributed to the overall ratings of some concerns.

**Table 2:**
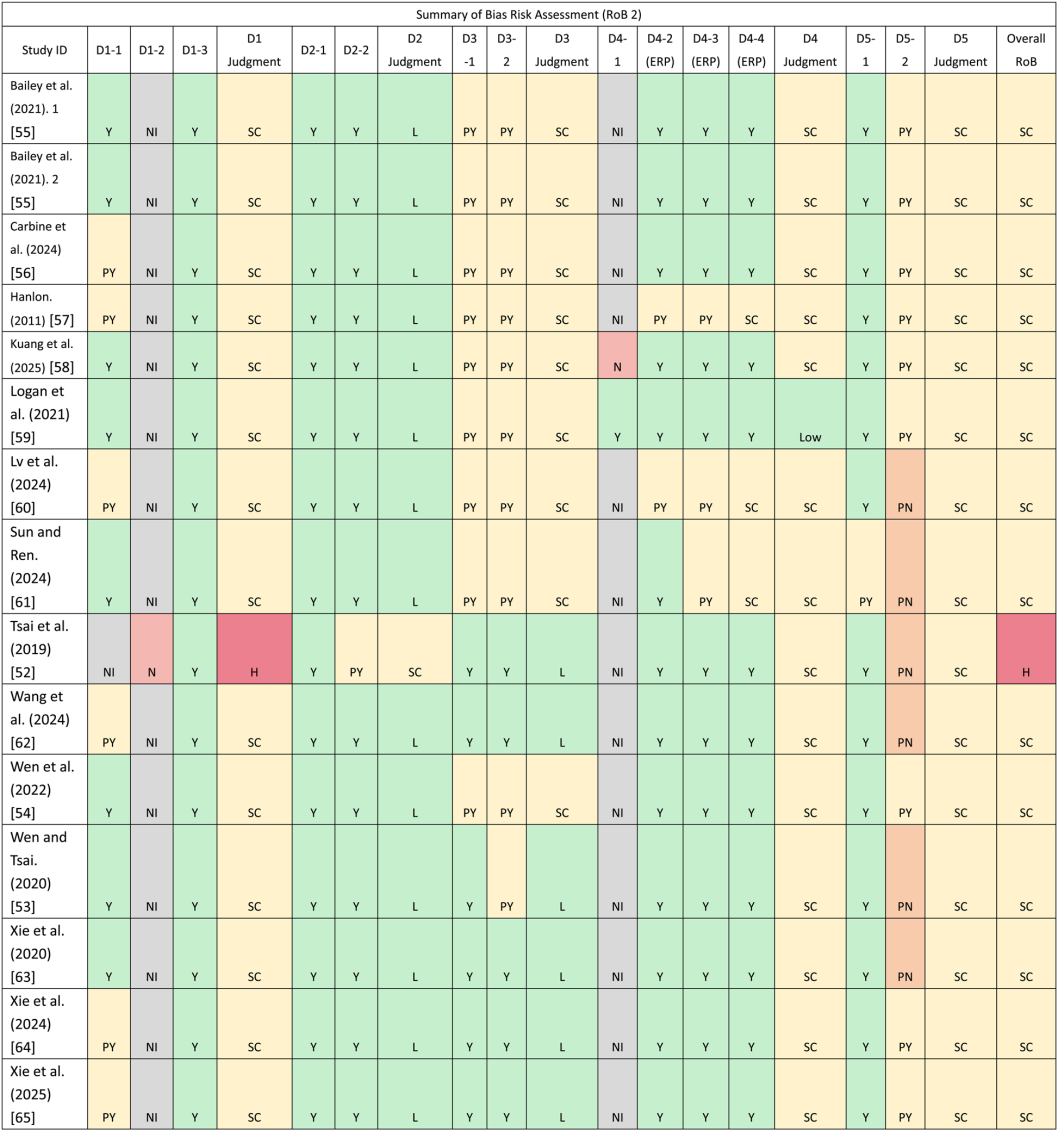
Summary of bias risk assessment. [55], [55–61], [52], [62], [54], [53], [63–65], [55, 61], [66], [62], [67], [68], [63, 65]

D1-1 refers to randomization, D1-2 refers to allocation concealment, D1-3 refers to baseline comparability; D2-1 refers to between-group analysis appropriate, D2-2 refers to exercise intervention sufficiently evaluated/reported; D3-1 refers to missingness acceptable, D3-2 refers to handling appropriate/balanced; D4-1 refers to assessor blinding, D4-2(ERP) refers to ERP acquisition/preprocessing adequate? D4-3(ERP) refers to ERP analysis/reporting adequate? D4-4(ERP) refers to ERP adequacy; D5-1 refers to point estimates with variability adequately reported, D5-2 refers to prespec/multiplicity control sufficient

Y (Yes): Clearly meets the criterion; evidence is sufficient with no material reservations. PY (Probably Yes): Largely meets the criterion; although reporting may be incomplete or not all details are specified, the procedures/text strongly suggest the criterion is met. PN (Probably No): Largely does not meet the criterion; while explicit contrary evidence may be absent, the procedures/text strongly suggest the criterion is not met. N (No): Clearly does not meet the criterion; there is direct evidence or a clear rationale indicating non-compliance. NI (No Information): Key information is not reported or cannot be determined. L (Low): The domain is judged at low risk of bias—any departures from ideal practice are unlikely to meaningfully affect the outcome. SC (Some concerns): There are some concerns that bias may be present in the domain, but not enough to rate it High. H (High): The domain is at high risk of bias—problems are likely to substantially affect the outcome, or there are multiple issues indicating serious risk. Overall study judgment (RoB 2 rule): Low only if all domains are Low; High if any domain is High (or multiple problematic domains); Some concerns otherwise (≥1 domain with Some concerns and none High)

For the trial judged as high risk of bias [66], the main concern was unclear allocation concealment (D1), with domain D1-2 again driving the overall high-risk judgement.

More detailed, item-level information is provided in *Supplement Table *S2 (Risk-of-bias details). Taken together, these limitations have the potential to influence the internal validity and certainty of the evidence; accordingly, we conducted leave-one-out sensitivity analyses for each primary outcome and incorporated these findings into our GRADE certainty ratings.

### ERP reporting quality summary

We summarized ERP reporting using a structured checklist aligned with RoB-2 domains D4 and D5. Most studies adequately described referencing (*n* = 11) and filtering (*n* = 11), artifact-handling procedures (ICA/rejection thresholds; *n* = 11), and sampling rate (*n* = 12); however, only one study [59] implemented assessor blinding, with most others rated NI (no information), consistent with the previously noted practical difficulties of implementing blinding procedures in exercise-intervention trials.

A priori time windows and ROIs (P3: centro-parietal; N2: fronto-central) were specified in all trials, and most trials reported the trial counts kept per condition (*n* = 12), but SNR/QC metrics were frequently rated No/not reported (*n* = 10). Multiplicity control (predefined windows, FDR/cluster or equivalent) was inconsistently documented. These reporting gaps map to D4 (measurement)—via incomplete SNR/analysis detail and missing blinding—and to D5 (selection of the reported result)—via limited prespecification/multiplicity information. Item-level results for each study are provided in *Supplement Table *S1 (ERP checklist) and were considered in interpretation; robustness was examined via leave-one-out analyses.

### Main Meta-analytic and moderator effects

#### Reaction Time (RT)

Pooled effect. As shown in Table 3, across 12 comparisons, random-effects pooling indicated a significant benefit of exercise on RT (benefit = faster responses, i.e., reduced control costs): Hedges’ g = − 0.303 (95% CI − 0.504 to − 0.103; Z = 2.96, *p* = 0.003), with substantial inconsistency (I² = 76.6%).

**Table 3:** Summary of overall and moderator analyses on RT

	Z/F, *p*, I²	#ES	ES and CI
Overall Effect	Z = 2.96**, *p*= 0.003,* I²=76.6%*	12	−0.303** [− 0.504, − 0.103]
BMI	F (1, 10) = 0.00004, *p* = 0.987, I²=78.46%		
Overweight		6	−0.333** [− 0.568, − 0.097]
Obesity		6	−0.336 [− 0.765, 0.093]
Gender	F (1, 10) = 2.62, *p* = 0.135, I²=72.26%		
Male only		6	-0.716** [− 1.246, -0.185]
Both		6	-0.178*** [− 0.265, -0.091]
Age	F (1, 10) = 0.18, *p* = 0.686, I²=78.46%		
≤ 18		1	-0.095 [− 0.483, 0.293]
19–35		11	-0.327** [− 0.543, -0.111]
Experimental Design	F (3, 8) = 0.40, *p* = 0.757, I²=76.01%		
Within, Post, Rand.		8	-0.207 [− 0.417, 0.002]
Within, Pre-Post, Rand.		2	-0.432 [− 1.229, 0.365]
Between, Pre-Post, Rand.		1	-0.095 [− 0.483, 0.293]
Between, Post, Rand.		1	-1.188** [− 1.899, -0.476]
Task Paradigm	F (2, 9) = 3.60, *p* = 0.071, I²=65.56%		
Go/nogo		3	-0.194** [− 0.317, -0.071]
Flanker		7	-0.246 [− 0.546, 0.053]
Others		2	-0.885*** [− 1.173, -0.597]
intervention type	F (1, 10) = 0.19, *p* = 0.673, I²=78.56%		
Acute		10	-0.271* [− 0.482, -0.061]
Training		2	-0.599 [− 1.666, 0.468]
Exercise Mode	F (3, 8) = 0.67, *p* = 0.595, I²=74.62%		
Aerobic		6	-0.182*** [− 0.269, -0.095]
Resistance		1	-0.825*** [− 1.140, -0.510]
HIIE		3	-0.877 [− 2.073, 0.319]
Combined		2	-0.599 [− 1.666, 0.468]
Exercise Intensity	F (1, 10) = 0.61, *p* = 0.453, I²=77.85%		
Moderate		6	-0.162 [− 0.418, 0.094]
High		6	-0.434** [− 0.734, -0.133]
Exercise Duration	F (3, 8) = 0.26, *p* = 0.855, I²=81.34%		
<30 min		3	-0.313 [− 0.893, 0.266]
30–39 min		4	-0.610 [− 1.348, 0.128]
40–60 min		3	-0.195*** [− 0.288, -0.102]
Long-term		2	-0.599 [− 1.666, 0.468]
Timing Before ERP Assessment	F (2, 7) = 0.04, *p* = 0.957, I²=78.77%		
≤ 15 min		6	-0.324 [− 0.771, 0.124]
15–30 min		2	-0.211*** [− 0.307, -0.115]
NI		2	-0.432 [− 1.229, 0.365]
Control Activity	F (2, 9) = 0.73, *p* = 0.507, I²=77.26%		
Baseline/assessment-only		7	-0.595* [− 1.054, -0.137]
Passive cognitive engagement		3	-0.195*** [− 0.288, -0.102]
Cognitive engagement		2	-0.054 [− 0.375, 0.266]

*p < 0.05; **p < 0.01; ***p< 0.001

**Fig. 2:**
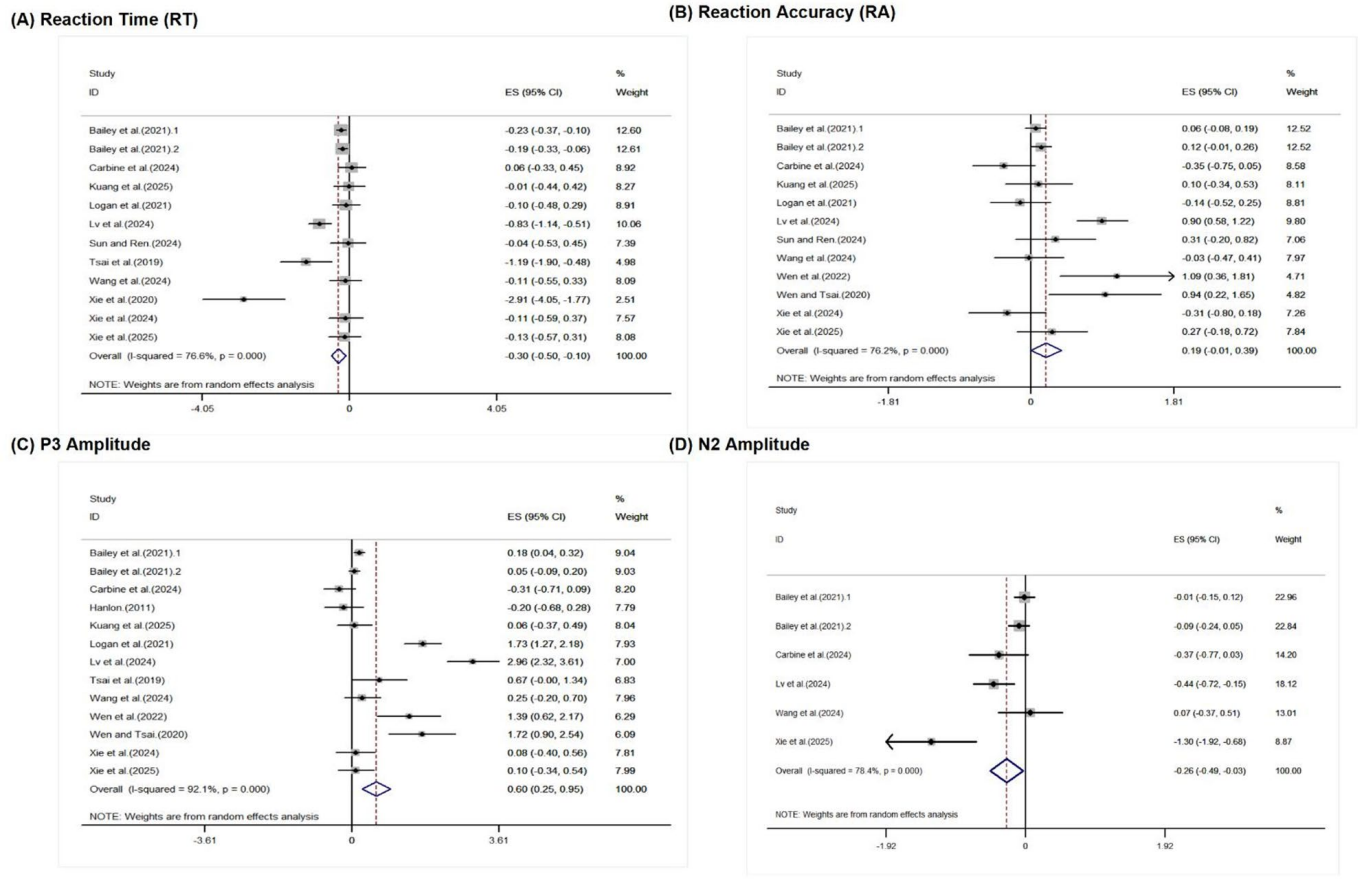
Forest plots of Hedges’ g for outcome measures (RT, RA, P3, and N2) across included studies. Each panel (**A**–**D**) shows study-level standardized effects (Hedges’ g) with 95% confidence intervals and the pooled random-effects estimate. Square sizes reflect inverse-variance weights; diamonds denote pooled effects

Heterogeneity and key subgroups. Given the substantial heterogeneity (I² = 76.6%), we applied random-effects models. The forest plot (Fig. 2-A) indicated considerable dispersion across studies. Prespecified subgroup analyses did not identify clear between-group differences (between-subgroup p-values ranged from 0.07 to 0.98), although task paradigm showed a borderline omnibus signal (F (2,9) = 3.60, *p* = 0.071). Within-subgroup inconsistency was low in some levels (e.g., mixed-sex samples, aerobic exercise, 40–60 min duration, and passive cognitive engagement controls; I² ≈ 0%), suggesting these study features may partially structure heterogeneity, whereas inconsistency remained substantial in others (e.g., overweight: I² ≈ 79%; high-intensity: I² ≈ 86%). Overall, residual heterogeneity remained notable; given the small number of studies in several strata and the largely non-significant between-group tests, these patterns are descriptive and should be interpreted cautiously.

Meta-regression. Meta-regression (REML with Knapp–Hartung inference) results were broadly consistent with the subgroup patterns, overall providing little evidence that the prespecified moderators explained between-study variability, although task-paradigm contrasts suggested potential differences. For task paradigm, although the omnibus test was only suggestive (F (2,9) = 3.60, *p* = 0.071), specific contrasts indicated larger (more negative) effects for Flanker versus Go/No-Go (b = − 0.691, SE = 0.272, *p* = 0.032) and for Others versus Flanker (b = − 0.705, SE = 0.268, *p* = 0.027). These contrasts should be interpreted cautiously given the small number of studies in some task categories. Full between-group tests, within-level pooled effects, and meta-regression coefficients (b, SE, t, p) are reported in *Supplement Table *S3* (Sheet1 RT)*.

#### Reaction accuracy (RA)

Pooled effect. As shown in Table 4, across 12 comparisons, random-effects pooling suggested a small improvement in RA favoring exercise, but the overall effect did not reach conventional statistical significance: Hedges’ g = 0.191 (95% CI − 0.006 to 0.387; Z = 1.90, *p* = 0.058), with substantial inconsistency (I² = 76.2%).

**Table 4:** Summary of overall and moderator analyses on RA

	Z/F, *p*, I²	#ES	ES and CI
Overall Effect	Z = 1.90, *p* = 0.058,* I²=76.2%*	12	0.191 [-0.006, 0.387]
BMI	F (1, 10) = 0.00, *p* = 0.997, I²=78.36%		
Overweight		5	0.203 [-0.078, 0.484]
Obesity		7	0.194 [-0.123, 0.512]
Gender	F (2, 9) = 4.00, *p* = 0.057, I²=67.76%		
Male only		4	0.225 [-0.331, 0.781]
Female only		2	1.009*** [0.499, 1.520]
Both		6	0.048 [-0.071, 0.167]
Age	F (1, 10) = 0.64, *p* = 0.442, I²=77.44%		
≤ 18		1	-0.138 [-0.523, 0.247]
19–35		11	0.223* [0.014, 0.432]
Experimental Design	F (2, 9) = 2.05, *p* = 0.185 I²=69.91%		
Within, Post, Rand.		7	0.044 [-0.085, 0.173]
Within, Pre-Post, Rand.		2	0.511 [-0.276, 1.297]
Between, Pre-Post, Rand.		3	0.583 [-0.284, 1.450]
Task Paradigm	F (3, 8) = 10.06, *p* = 0.004, I²=18.28%		
Go/nogo		3	-0.003 [-0.281, 0.276]
Flanker		6	0.075 [-0.040, 0.190]
Stroop		2	1.009*** [0.499, 1.520]
Others		1	0.899*** [0.576, 1.222]
intervention type	F (1, 10) = 0.19, *p* = 0.672, I²=78.39%		
Acute		10	0.176 [-0.029, 0.378]
Training		2	0.075 [-0.433, 1.629]
Exercise Mode	F (3, 8) = 2.35, *p* = 0.148, I²=62.27%		
Aerobic		6	0.065 [-0.039, 0.169]
Resistance		1	0.899*** [0.576, 1.222]
HIIE		2	-0.003 [-0.607, 0.602]
Combined		3	0.583 [-0.284, 1.450]
Exercise Intensity	F (1, 10) = 0.01, *p* = 0.914, I²=78.17%		
Moderate		7	0.183 [-0.143, 0.509]
High		5	0.224 [-0.053, 0.501]
Exercise Duration	F (3, 8) = 0.65, *p* = 0.606, I²=74.77%		
<30 min		3	0.454 [-0.072, 0.981]
30–39 min		4	0.162 [-0.273, 0.598]
40–60 min		3	0.032 [-0.133, 0.197]
Long-term		2	0.433 [-0.763, 1.629]
Timing Before ERP Assessment	F (2, 7) = 1.58, *p* = 0.271, I²=67.22%		
≤ 15 min		5	-0.034 [-0.304, 0.236]
15–30 min		3	0.147 [-0.050, 0.343]
NI		2	0.511 [-0.276, 1.297]
Control Activity	F (2, 9) = 2.06, *p* = 0.183, I²=72.46%		
Baseline/assessment-only		7	0.437* [0.062, 0.811]
Passive cognitive engagement		3	0.032 [-0.133, 0.197]
Cognitive engagement		2	-0.088 [-0.482, 0.306]

*p < 0.05; **p < 0.01; ***p< 0.001

Heterogeneity and key subgroups. Given the high I², the forest plot (Fig. 2-B) showed wide dispersion across studies. Most prespecified subgroup analyses did not detect clear between-group differences. Two factors, however, showed comparatively stronger signals: task paradigm demonstrated a significant between-subgroup difference (F (3,8) = 10.06, *p* = 0.004) with relatively low heterogeneity at the factor level (I² = 18.28%), and gender showed a near-significant omnibus signal (F (2,9) = 4.00, *p* = 0.057). Within-subgroup inconsistency was low in some levels (e.g., mixed-sex samples, I² ≈ 27%; Flanker paradigm, I² ≈ 1.5%; aerobic exercise, I² ≈ 14.3%), suggesting these study features may partially structure heterogeneity, whereas inconsistency remained substantial in others (e.g., overweight, I² ≈ 86%; high-intensity exercise, I² ≈ 85%). Overall, residual heterogeneity remained substantial; given the small number of studies in several strata, these patterns are descriptive and should be regarded as exploratory and interpreted cautiously.

Meta-regression. Meta-regression (REML with Knapp–Hartung inference) results were broadly consistent with the subgroup patterns, overall providing limited evidence that the prespecified moderators explained between-study variability, although task-paradigm contrasts suggested potential differences. Specifically, relative to the Go/No-Go paradigm, Stroop showed a larger RA benefit (b = 0.993, SE = 0.284, *p* = 0.008), and, relative to Flanker, Stroop exhibited a further increase in effect size (b = 0.959, SE = 0.280, *p* = 0.009). In addition, one exercise-mode contrast reached nominal significance (*p* = 0.049) and one gender contrast was significant (*p* = 0.021), but these findings should be interpreted cautiously given sparse data in some categories (including strata with k = 1–2). Full between-group tests, within-level pooled effects, and meta-regression coefficients (b, SE, t, p) are reported in *Supplement Table *S3* (Sheet2 RA)*.

#### P3 amplitude

Pooled effect. As shown in Table 5, across 13 comparisons, random-effects pooling indicated a significant benefit of exercise on P3 amplitude: Hedges’ g = 0.602 (95% CI 0.254 to 0.949; Z = 3.39, *p* = 0.001), with high inconsistency (I² = 92.1%).

**Table 5:** Summary of overall and moderator analyses on P3 amplitude

	Z/F, *p*, I²	#ES	ES and CI
Overall Effect	Z = 3.39**, *p* = 0.001,* I²=92.1%*	13	0.602** [0.254, 0.949]
BMI	F (1, 11) = 0.14, *p* = 0.718, I²=92.02%		
Overweight		6	0.478* [0.007, 0.949]
Obesity		7	0.721* [0.147, 1.296]
Gender	F (2, 10) = 0.40, *p* = 0.680, I²=92.80%		
Male only		5	0.796 [-0.136, 1.727]
Female only		3	0.938 [-0.361, 2.238]
Both		5	0.312 [-0.085, 0.709]
Age	F (1, 11) = 1.44, *p* = 0.255, I²=89.95%		
≤ 18		1	1.725*** [1.270, 2.180]
19–35		12	0.486** [0.166, 0.807]
Experimental Design	F (3, 9) = 4.45, *p* = 0.035, I²=85.61%		
Within, Post, Rand.		7	0.072 [-0.045, 0.190]
Within, Pre-Post, Rand.		2	1.502 [-1.345, 4.349]
Between, Pre-Post, Rand.		3	1.655*** [1.301, 2.009]
Between, Post, Rand.		1	0.672 [-0.001, 1.344]
Task Paradigm	F (3, 9) = 1.48, *p* = 0.285, I²=92.07%		
Go/nogo		3	0.026 [-0.270, 0.322]
Flanker		5	0.424 [-0.131, 0.979]
Stroop		2	1.548*** [0.985, 2.112]
Others		3	1.138 [-0.745, 3.022]
intervention type	F (1, 11) = 1.69, *p* = 0.220, I²=89.45%		
Acute		10	0.399* [0.062, 0.737]
Training		3	1.293*** [0.643, 1.944]
Exercise Mode	F (3, 9) = 30.90, *p* = 0.000, I²=38.59%		
Aerobic		7	0.071 [-0.046, 0.187]
Resistance		1	2.964*** [2.324, 3.605]
HIIE		1	0.082 [-0.397, 0.562]
Combined		4	1.389*** [0.883, 1.895]
Exercise Intensity	F (1, 11) = 0.11, *p* = 0.748, I²=92.56%		
Moderate		9	0.562* [0.051, 1.073]
High		4	0.707* [0.121, 1.294]
Exercise Duration	F (3, 9) = 2.12, *p* = 0.168, I²=88.90%		
<30 min		2	1.502 [-1.345, 4.349]
30–39 min		4	0.444 [-0.101, 0.990]
40–60 min		4	0.022 [-0.156, 0.200]
Long-term		3	1.293*** [0.643, 1.944]
Timing Before ERP Assessment	F (3, 6) = 1.09, *p* = 0.423, I²=91.92%		
≤ 15 min		4	0.013 [-0.236, 0.262]
15–30 min		3	0.331 [-0.024, 0.686]
≥ 31 min		1	0.201 [-0.685, 0.283]
NI		2	1.502 [-1.345, 4.349]
Control Activity	F (2, 10) = 2.14, *p* = 0.169, I²=90.41%		
Baseline/assessment-only		8	1.058 [0.321, 1.796]
Passive cognitive engagement		3	0.048 [-0.140, 0.237]
Cognitive engagement		2	0.070 [-0.251, 0.390]

*p < 0.05; **p < 0.01; ***p< 0.001

Heterogeneity and key subgroups. Visual inspection of the forest plot (Fig. 2-C) indicated wide dispersion of effect estimates across studies. Several prespecified moderators helped structure this variability. Between-subgroup tests were significant for experimental design (F (3,9) = 4.45, *p* = 0.035) and exercise mode (F (3,9) = 30.90, *p* < 0.001), and the factor-level heterogeneity for exercise mode was markedly lower (I² = 38.6%). Within-subgroup inconsistency decreased to low or near-zero in some strata (e.g., between-group pre–post randomized, I² ≈ 0%; aerobic exercise, I² ≈ 22%; ≤15 min test interval, I² ≈ 22%), but remained substantial in others (e.g., overweight, I² ≈ 94%; high-intensity, I² ≈ 96%; acute exercise, I² ≈ 91%). Accordingly, meaningful residual heterogeneity remained.

Meta-regression. Meta-regression (REML with Knapp–Hartung inference) results were broadly consistent with the subgroup patterns, with statistically significant contrasts for study design and exercise mode. Relative to the within-subject posttest randomized reference category (Within-Post-Rand), larger P3 effects were observed for within-subject pre–post randomized (Within-Pre-Post-Rand; b = 1.401, SE = 0.564, *p* = 0.035) and between-group pre–post randomized designs (Between-Pre-Post-Rand; b = 1.595, SE = 0.500, *p* = 0.011). Relative to aerobic exercise, P3 gains were substantially larger for resistance training (b = 2.906, SE = 0.392, *p* < 0.001) and combined exercise (b = 1.371, SE = 0.206, *p* < 0.001); resistance training also exceeded HIIE (b = 2.882, SE = 0.487, *p* < 0.001). Other contrasts (e.g., task paradigm and control activity) were not statistically significant. Full between-group tests, within-level pooled effects, and meta-regression coefficients (b, SE, t, p) are reported in *Supplement Table *S3* (Sheet3 P3)*.

#### N2 amplitude

Pooled effect. As shown in Table 6, across 6 comparisons, random-effects pooling indicated a significant benefit of exercise on N2 amplitude (coded as greater absolute negativity, i.e., more negative values): Hedges’ g = − 0.262 (95% CI − 0.492 to − 0.032; Z = 2.24, *p* = 0.025), with substantial inconsistency (I² = 78.4%).

**Table 6:** Summary of overall and moderator analyses on N2 amplitude

	Z/F, *p*, I²	#ES	ES and CI
Overall Effect	Z = 2.24*, *p* = 0.025,* I²=78.4%*	6	-0.262* [-0.492, -0.032]
BMI	F (1, 4) = 0.57, *p* = 0.494, I²=80.87%		
Overweight		4	-0.173 [-0.349, 0.004]
Obesity		2	-0.596 [-1.930, 0.738]
Gender	F (1, 4) = 0.91, *p* = 0.395, I²=73.69%		
Male only		3	-0.515 [-1.137, 0.106]
Both		3	-0.081 [-0.206, 0.044]
Experimental Design	F (1, 4) = 0.08, *p* = 0.793, I²=77.81%		
Within, Post, Rand.		5	-0.221 [-0.469, 0.028]
Within, Pre-Post, Rand.		1	-0.437** [-0.720, -0.153]
Task Paradigm	F (2, 3) = 0.52, *p* = 0.640, I²=83.29%		
Go/nogo		3	-0.494 [-1.116, 0.128]
Flanker		2	-0.078 [-0.213, 0.058]
Others		1	-0.437** [-0.720, -0.153]
Exercise Mode	F (1, 4) = 0.08, *p* = 0.793, I²=77.81%		
Aerobic		5	-0.221 [-0.469, 0.028]
Resistance		1	-0.437** [-0.720, -0.153]
Exercise Intensity	F (1, 4) = 0.62, *p* = 0.475, I²=79.17%		
Moderate		3	-0.499 [-1.185, 0.187]
High		3	-0.142 [-0.330, 0.046]
Exercise Duration	F (2, 3) = 0.36, *p* = 0.726, I²=80.22%		
<30 min		1	-0.437** [-0.720, -0.153]
30–39 min		2	-0.596 [-1.930, 0.738]
40–60 min		3	-0.081 [-0.206, 0.044]
Timing Before ERP Assessment	F (2, 3) = 0.46, *p* = 0.668, I²=76.76%		
≤ 15 min		3	-0.499 [-1.185, 0.187]
15–30 min		2	-0.052 [-0.151, 0.147]
NI		1	-0.437** [-0.720, -0.153]
Control Activity	F (1, 4) = 0.91, *p* = 0.395, I²=73.69%		
Baseline/assessment-only		3	-0.515 [-1.137, 0.106]
Passive cognitive engagement		3	-0.081 [-0.206, 0.044]

*p < 0.05; **p < 0.01; ***p< 0.001

Heterogeneity and key subgroups. The forest plot (Fig. 2-D) indicated wide dispersion of effect estimates across studies. Given the relatively small evidence base for N2 (overall k = 6) and the fact that several strata contained only a single comparison (yielding I² = NA), some prespecified moderators overlapped across levels and no statistically significant between-group differences were detected. Within strata, pooled effects were generally negative (favoring exercise). Within-subgroup inconsistency was low in some levels (e.g., Flanker paradigm, I² ≈ 0%; mixed-sex samples, I² ≈ 30%; 40–60 min duration, I² ≈ 30%; passive cognitive engagement controls, I² ≈ 30%), whereas heterogeneity remained substantial in others (e.g., moderate intensity, I² ≈ 84%; baseline/assessment-only controls, I² ≈ 84%; overweight category, I² ≈ 65%). Overall, meaningful residual heterogeneity remained; given the small number of studies per stratum (including some k = 1 levels), subgroup patterns should be interpreted cautiously and regarded as descriptive.

Meta-regression. Meta-regression (REML with Knapp–Hartung inference) was broadly consistent with the subgroup results, providing limited evidence that prespecified moderators explained between-study variability. No statistically significant associations were observed for gender (b = 0.352, SE = 0.369, *p* = 0.395), experimental design (b = − 0.152, SE = 0.543, *p* = 0.793), exercise mode (b = − 0.152, SE = 0.543, *p* = 0.793), task paradigm or control activity. Full between-group tests, within-level pooled effects, and meta-regression coefficients (b, SE, t, p) are reported in *Supplement Table *S3* (Sheet4 N2)*.

### Sensitivity, publication bias, and certainty of evidence (GRADE)

#### Sensitivity analysis

**Fig. 3:**
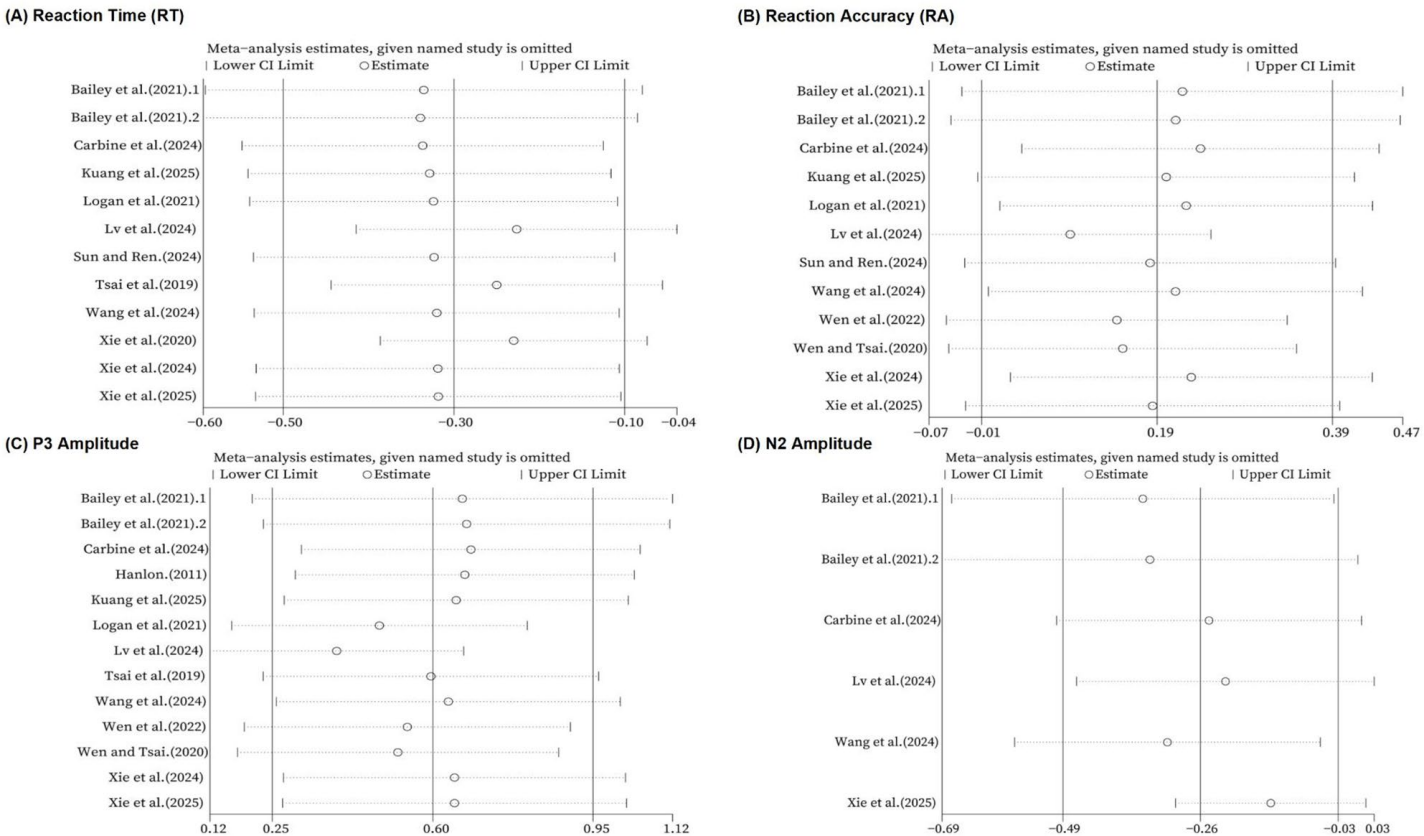
Leave-one-out sensitivity analyses for outcome measures (RT, RA, P3, and N2). Each panel (**A**–**D**) shows the pooled effect (Hedges’ g) and 95% confidence interval recalculated after excluding one study at a time (random-effects model)

To evaluate robustness, we conducted leave-one-out analyses for all four outcomes (Fig. 3.), following Cochrane Handbook recommendations for sensitivity checks in meta-analysis [29, 50]. The pooled RT effect remained stable in both magnitude and direction, and no single study altered the statistical significance of the overall finding. Although the overall pooled RA effect was borderline and did not reach conventional statistical significance (g ≈ 0.19; 95% CI included zero), omitting three studies [56, 62,64 ] yielded confidence intervals that no longer crossed zero, indicating comparatively lower stability for RA. The pooled effect for P3 amplitude remained consistently positive and statistically significant across all leave-one-out iterations. For N2 amplitude, leave-one-out analyses showed a consistent direction of effect, but statistical significance was sensitive to the omission of several studies [56, 60, 65], after which the 95% confidence interval crossed zero; this pattern likely reflects the small evidence base (k = 6) and greater vulnerability to single-study influence.

Sensitivity to the assumed within-person correlation (r). Because most included trials used within-subject/crossover contrasts, we prespecified sensitivity analyses varying the assumed within-person correlation used to derive the SD of paired differences (*r* = 0.3/0.5/0.7, details are in *Supplement Table *S4). Across all outcomes, the direction of effects and substantive inferences were unchanged. For RT, pooled effects remained significantly negative and of similar magnitude. For P3, pooled effects remained significantly positive. For N2, pooled effects remained significantly negative. In contrast, RA remained borderline across r values, consistent with the primary analysis. Notably, heterogeneity remained substantial across r assumptions (I² ≈ 72–92%), indicating that the conclusions are robust to plausible correlation values but that between-study variability persists.

Excluding single-condition extractions. Finally, because two studies [60, 68] contributed effects based on single-condition values (rather than contrast-based indices), we repeated the primary analyses after excluding these studies. Although pooled effect sizes attenuated slightly, the overall direction of effects and the position of the confidence intervals were unchanged for RT, RA, P3, and N2, indicating that the main conclusions were not driven by the small subset of single-condition extractions.

Overall, the sensitivity analyses support the robustness of the main conclusions for RT and P3 amplitude, indicate that evidence for RA is borderline and study-sensitive, and suggest that N2 effects are directionally consistent but precision-limited due to the small number of available comparisons.

#### Publication bias / small-study effects

**Fig. 4:**
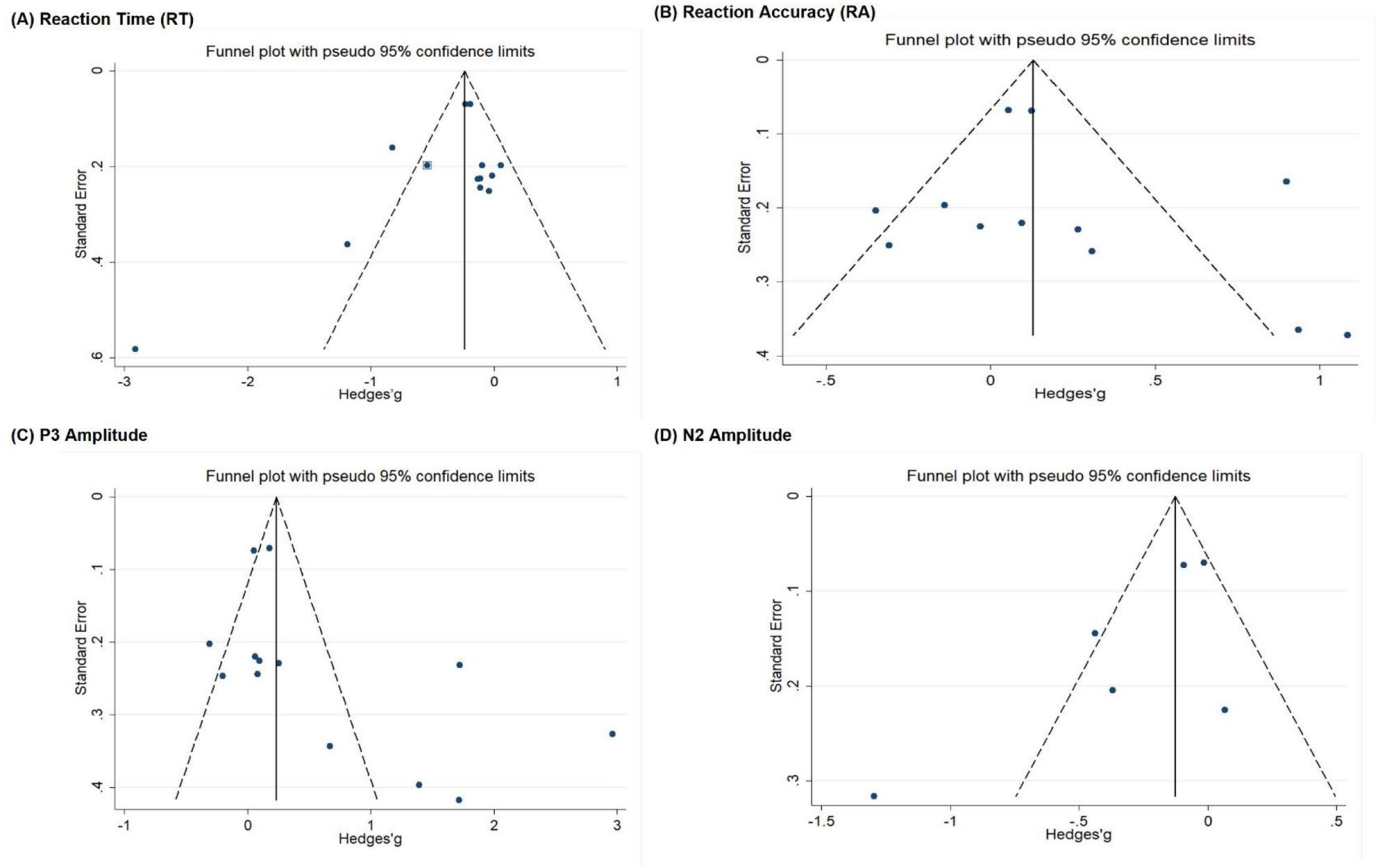
Funnel plots assessing small-study and publication bias for outcome measures (RT, RA, P3, and N2). Each panel (A–D) plots the study standard error against effect size (Hedges’ g). The vertical reference line marks the pooled random-effects estimate, and the dashed pseudo–95% confidence limits indicate the expected dispersion in the absence of small-study effects/publication bias

Potential small-study and reporting biases were assessed with funnel plots and Egger’s regression [51], interpreted alongside heterogeneity and power considerations [29, 47, 50]. Funnel plots for each outcome (Fig. 4.) appeared roughly symmetric. Egger’s tests showed no statistical evidence of small-study bias for RT (*p* = 0.291), RA (*p* = 0.437), P3 (*p* = 0.076), or N2 (*p* = 0.107; note low power at k = 6). We additionally applied the trim-and-fill method [52]. Across the four outcomes, only RT suggested potential missingness: under the random-effects model, the procedure imputed one potentially missing study (Fig. 4. A). After filling, the pooled RT effect changed from g = − 0.303 (95% CI − 0.504 to − 0.103) to g = − 0.341 (95% CI − 0.538 to − 0.145), with the direction unchanged and the effect remaining statistically significant (z = − 3.40, *p* = 0.001), suggesting that the RT conclusion was robust even after accounting for potential publication bias. Taken together, there was no clear evidence of publication or reporting bias, acknowledging limited power for asymmetry tests in small sets—especially for N2.

#### Certainty of evidence (GRADE)

Using GRADE—where RCTs start at High—we rated the certainty for each primary outcome [53, 54] (see *Supplement Table *S5,* Summary-of-Findings*):

Reaction Time (RT): Moderate certainty, downgraded for risk of bias (frequent Some concerns, especially ERP measurement/reporting) and inconsistency (I² = 76.6%). Reaction Accuracy (RA): Low certainty, downgraded for risk of bias and inconsistency (I² = 76.2%), and for imprecision (confidence interval crossing zero). P3 amplitude: Moderate certainty, downgraded for risk of bias and inconsistency (I² = 92.1%); overall precision was acceptable. N2 amplitude: Low certainty, downgraded for risk of bias, inconsistency (I² = 78.4%), and imprecision (small k and sample size; CI close to the null). Overall certainty ranges from moderate (RT, P3) to low (RA, N2), indicating that additional well-reported, adequately powered RCTs are needed to strengthen these estimates.

## Discussion

### Principal findings

Across overweight/obese samples, exercise was associated with faster reaction time (RT; Hedges’ g = − 0.303, 95% CI − 0.504 to − 0.103) and larger P3 amplitudes (g = 0.602, 95% CI 0.254 to 0.949). Reaction accuracy showed a borderline improvement (g = 0.191, 95% CI − 0.006 to 0.387; CI crossing zero), and N2 became more negative (g = − 0.262, 95% CI − 0.492 to − 0.032). Heterogeneity was substantial (I² ≈ 70–90%). Prespecified moderators explained limited dispersion overall; however, P3 varied by exercise mode and study design, and RA varied by task paradigm. Leave-one-out analyses indicated robust findings for RT and P3, but greater study sensitivity for RA and N2. Funnel/Egger tests suggested no clear small-study effects; trim-and-fill imputed one study for RT, with the adjusted effect remaining significant. By GRADE, certainty was moderate for RT and P3 and low for RA and N2, driven by risk of bias, inconsistency, and (for RA/N2) imprecision.

### Interpretation in the context of prior evidence

#### Behavioral outcomes (RT, RA)

Our RT finding is consistent with quantitative syntheses showing that a single bout of exercise yields small but reliable benefits on executive-task performance, with effects often more apparent for speeded outcomes and shaped by task demands and exercise parameters (e.g., intensity and post-exercise timing) [42, 69]. Larger-scale reviews similarly suggest that cognitive benefits vary with FITT-VP dose attributes across populations [70–73]. In contrast, accuracy effects are typically smaller and less stable across paradigms and comparator conditions, which aligns with accounts proposing that exercise may preferentially shift efficiency or response thresholds, improving speed more readily than correctness [42, 69].

#### ERP outcomes (P3, N2)

Our ERP results broadly converge with prior evidence that exercise can enhance neural indices relevant to executive control. The post-exercise increase in P3 amplitude is consistent with reviews reporting P3 augmentation after acute moderate-to-vigorous exercise, often moderated by dose characteristics and task requirements [18, 42, 74, 75]. The more negative N2 observed here is directionally consistent with reports suggesting strengthened conflict monitoring/inhibitory processing following exercise; however, N2 effects are less consistently reported and the evidence base is smaller, which plausibly contributes to greater uncertainty and sensitivity to design features (e.g., timing, control activity) [75, 76]. aken together, the pattern of P3 enhancement and greater N2 negativity is compatible with exercise-related improvements in attentional allocation and control signaling, while the relative instability of RA underscores that behavioral improvements may not uniformly generalize across performance indices [72, 77].

#### Overweight/obese context and contribution

Evidence specific to individuals with elevated BMI remains comparatively sparse and heterogeneous, yet available studies suggest that the canonical RT benefit of exercise extends to overweight/obese samples, whereas accuracy gains are generally smaller and less stable [64, 68, 78]. ERP findings in these cohorts—such as post-exercise increases in P3 and task-dependent N2 modulation—provide converging neural support for changes in attentional allocation and conflict processing [64, 68]. Distinctively, the present review isolates overweight/obese cohorts and integrates behavioral outcomes with ERP indices (P3, N2), while harmonizing key design features (exercise dose, exercise-to-ERP timing in acute trials, and control-activity taxonomy). This synthesis helps clarify where benefits appear most consistently despite variability in study designs and reporting quality.

### Potential mechanisms

Two complementary pathways plausibly account for the observed pattern—faster RT, larger P3, and a more negative N2 following exercise—while recognizing that RA gains were small and less consistent. (i) Acute neurotransmitter regulation and arousal mechanisms. A single bout of exercise can transiently elevate catecholaminergic activity (noradrenaline/dopamine), increase cortical arousal, and improve signal-to-noise within frontoparietal control networks, thereby facilitating executive control engagement in the short term [27]. A central candidate is the LC–NE system: phasic LC–NE activity is theorized to optimize task engagement (“adaptive gain”) and is closely linked to P3 generation [79, 80]. Within this framework, a larger P3 reflects enhanced attentional allocation/context updating [7], whereas a more negative N2 is consistent with strengthened conflict monitoring and/or inhibitory control signals (e.g., ACC-related processes) [81]. These mechanisms also provide a parsimonious explanation for why effects tended to be clearer under protocols that likely induce higher acute arousal (e.g., HIIE or resistance) and within favorable post-exercise timing windows, aligning with the stronger RT and P3 gains observed in our moderator analyses [79, 80, 82].

(ii) Training-related neurobiological adaptations and neuroplasticity. With multi-week programs, improvements in cardiorespiratory and muscular fitness, enhanced cerebrovascular function, and neurotrophic signaling (e.g., BDNF) may provide a longer-acting substrate for executive-control benefits [83, 84]. Such adaptations could sustain or amplify acute neuromodulatory effects, offering a plausible route by which chronic exercise maintains P3 enhancement and N2 modulation alongside modest behavioral improvements. Finally, task characteristics (e.g., inhibition vs. conflict paradigms) can shift the balance between attention allocation and conflict processing, thereby modulating P3/N2 sensitivity, and control-condition features that capture attention/expectancy may partially attenuate between-group contrasts—patterns that are consistent with the moderator structure observed across outcomes.

### Moderators

#### Exercise mode, intensity, and duration

Mode. Across outcomes, exercise mode emerged as one of the more informative moderators, with higher-arousal modes (e.g., resistance training and HIIE) tending to show more consistent benefits than pure aerobic exercise in several outcomes. This pattern aligns with syntheses suggesting that protocols inducing greater acute neuro-metabolic load and catecholaminergic arousal may translate—within short post-exercise windows—into larger P3 amplitudes and faster RT [18, 69, 85]. At the same time, mode comparisons should be interpreted cautiously because mode often covaries with task paradigm, participant characteristics, and control-condition design, and k was uneven across strata [18, 72, 85].

Duration. Duration-related patterns were consistent with prior reports of a non-linear (e.g., J- or inverted-U) relationship between exercise dose and executive performance, in which longer sessions may introduce fatigue or competing physiological stressors that offset attentional benefits [69, 85]. In addition, heterogeneous post-exercise assessment timing and control activities can obscure duration effects; accordingly, future trials should preregister duration strata and harmonize control conditions to improve comparability.

Intensity. The available evidence did not indicate clear differences between moderate- and high-intensity interventions, which is consistent with the view that intensity effects depend on task demands, assessment timing, and individual responsiveness (e.g., baseline fitness or metabolic status) [18, 75]. Nevertheless, point estimates tended to be larger in the high-intensity category, tentatively suggesting that higher intensities may elicit stronger arousal and metabolic signaling. Importantly, the current evidence does not support a simple “higher is better” rule; rather, it is more consistent with a context-dependent and potentially non-linear dose–response pattern [69, 85].

Intervention type (acute vs. training). The available evidence did not show clear differences between acute and training interventions, likely due to the small number of training comparisons (k = 3) and high heterogeneity. However, given distinct underlying mechanisms—and the possibility that training produces more durable benefits via fitness- and neuroplasticity-related pathways [10, 11, 72]—this null finding should be interpreted cautiously. Notably, point estimates tended to be larger for training than for acute interventions, particularly for RT and P3, suggesting potentially more sustained or cumulative improvements that warrant confirmation in adequately powered studies.

#### Timing before ERP assessment and experimental design

Timing before ERP assessment. Prior syntheses indicate that neurocognitive benefits are often greatest within ~ 10–30 min post-exercise and depend on task demands and dose [18, 42, 74, 75], In our analysis, timing did not yield a significant between-group difference, although point estimates were often larger in the 15–30 min stratum, suggesting potentially more stable effects in this window with attenuation thereafter—consistent with transient arousal/catecholaminergic mechanisms. Because timing strata were uneven and covaried with mode and task paradigm, this pattern should be interpreted as exploratory.

Experimental design. Study design contributed to variability, particularly for ERP outcomes. Designs with baseline assessment (pre–post) and/or between-group comparisons may yield more stable estimates by accounting for baseline differences and time effects, whereas post-only within-subject comparisons can be more susceptible to order/practice effects and incomplete washout in crossover settings. Consistent with methodological guidance, baseline-adjusted approaches (e.g., change scores or ANCOVA) can improve precision in randomized and crossover designs when carryover is adequately controlled [29, 86–88]. These design features may influence both behavioral contrasts and ERP estimates through differences in internal validity and measurement precision [29, 74]. Accordingly, design was retained as an exploratory moderator, and design-related findings—especially with sparse strata—should be interpreted cautiously.

#### Participant factors (BMI, age, gender)

Across outcomes, the available evidence does not suggest that exercise-related cognitive/ERP benefits are confined to a single BMI category. RT benefits and P3 augmentation appeared in both overweight and obesity strata, while N2 showed only a tentative tendency toward larger (more negative) changes in obesity. These tendencies are biologically plausible given BMI-related executive-control vulnerabilities and vascular/metabolic constraints (e.g., insulin resistance, low-grade inflammation) [73, 89], but current subgroup sizes are small and heterogeneous, limiting inference.

Age moderation was largely indeterminate because most comparisons were concentrated in young adults, with sparse representation of adolescents and older adults. Prior syntheses indicate that acute exercise reliably improves speeded executive performance across the lifespan, whereas accuracy effects are more variable and task dependent [14, 69]. Gender-related patterns were similarly suggestive: male-only cohorts tended to show larger speedups, whereas female-only samples sometimes showed modest accuracy advantages, potentially reflecting sex-linked differences in neuroendocrine and catecholaminergic dynamics [90]. Because sex composition covaried with protocol choices (including mode and intensity), causal interpretation should remain cautious. Overall, participant moderators in this dataset are best viewed as hypothesis generating.

#### Control-activity class and implications for expectancy/attention

Control-condition design systematically shaped observed effects and is a critical interpretive lens. Across outcomes, baseline/assessment-only controls tended to yield clearer contrasts, whereas more active sedentary controls—particularly passive reading or similar engagement—could dilute between-group differences by absorbing attention, altering expectancy, or inducing mild cognitive arousal (“active-control dilution”) [91–93]. This principle is consistent with broader methodological guidance that active controls may be necessary to manage nonspecific effects yet can attenuate observed contrasts [91, 92]. From an ERP standpoint, P3 is highly sensitive to attentional allocation [7]; thus, attention-capturing controls can narrow P3 differences even when exercise produces genuine neurocognitive benefits. In practice, future trials should preregister and justify control categories, include brief checks of expectancy/engagement where feasible, and report control-specific estimates to facilitate cross-study comparability.

#### Task paradigm

Task demands also contributed to variability, though signals differed by outcome. In our dataset, task paradigm showed clearer between-group structure for RA, with some paradigms exhibiting larger accuracy gains than others, whereas paradigm differences for RT were more suggestive overall. For P3 and N2, between-paradigm differences were not consistently detected after Knapp–Hartung adjustment, likely reflecting limited and imbalanced k (especially for N2) and heterogeneity within “paradigm” labels (e.g., variations in stimulus mappings and difficulty). Mechanistically, Stroop/Flanker tasks impose higher selective-attention and conflict-monitoring demands that map onto P3 (attentional allocation/context updating) and N2 (conflict/inhibitory control), whereas Go/No-Go emphasizes response inhibition with less sustained context updating [69, 85, 94]; thus, protocol–task alignment may influence whether exercise effects manifest more consistently in speed (RT), resource allocation (P3), or conflict processing (N2) [7, 95, 96]. In sum, paradigm-related findings should be regarded as exploratory, and future studies should adopt harmonized task implementations and contrast definitions to improve interpretability across trials.

### ERP methodological considerations

#### Reporting quality against the checklist

ERP reporting quality varied across trials. Basic settings (e.g., reference and filtering) and artifact handling were often described, but key details that can materially influence component estimation were frequently under-specified (e.g., rejection thresholds, ICA decision rules, re-referencing steps). Trial retention was not consistently reported in precision-relevant ways (e.g., per-condition usable trials and exclusion rules). Time windows and ROIs were commonly stated for P3 and N2, but deviations were seldom justified, and multiplicity control and assessor blinding/scripted pipelines were rarely documented. Moreover, only a subset of studies reported complete estimands required for synthesis (including variance information and, for crossover designs, within-person correlations). These gaps are inconsistent with contemporary ERP reporting guidance and may increase analytic flexibility and measurement noise [23, 97–99].

#### Mapping to RoB-2 (D4/D5) and implications for certainty

These limitations map directly onto RoB-2 domains relevant to ERP outcomes. Incomplete specification of preprocessing and trial-retention detail and unclear analytic windows/ROIs raise concerns under D4 (measurement), while incomplete variability reporting and inconsistent multiplicity control increase concerns under D5 (selection of the reported result). Accordingly, ERP-specific “No information” judgments contributed to “Some concerns” ratings and informed GRADE downgrades for risk of bias and inconsistency. This highlights the need for prespecified ERP analyses, standardized pipelines, and complete reporting (COBIDAS-MEEG) to improve reproducibility and certainty [25, 41, 98].

#### Recommendations for future ERP reporting

Drawing on the methodological shortcomings observed across included trials and current ERP best-practice guidance [23, 25, 41, 97–99], we propose the following recommendations to mitigate D4/D5 concerns, reduce heterogeneity, and enhance the reproducibility and certainty of future syntheses:

First, ERP analyses should be prespecified (conditions, components, ROIs/time windows, and mean vs. peak metrics), with clear rules for alternative windows if needed. Second, studies should report acquisition and preprocessing transparently (reference, sampling, filtering, epoch/baseline definitions, and explicit artifact/ICA criteria). Third, trials should provide trial-retention and precision-relevant quantities (per-condition usable trials, exclusion rules, variance estimates) and, for crossover designs, the within-person correlation (or sufficient information to derive it), enabling reproducible effect-size estimation. Where multiple windows/ROIs are explored, appropriate multiplicity control and clear separation of confirmatory versus exploratory analyses are required. Detailed checklist items can be provided in the Supplement.

### Clinical and public-health implications

#### Implications for exercise prescription in overweight/obesity

From an applied perspective, the most consistent signals were improvements in processing speed (RT) and enhanced attentional allocation (P3), whereas accuracy effects were smaller and less stable and N2 evidence was limited. Accordingly, exercise may be most defensible as an adjunct strategy to support executive-control efficiency in individuals with elevated BMI, particularly when interventions are designed to engage cognitive control shortly after exercise. These findings are compatible with exercise-prescription frameworks emphasizing FITT-VP principles and individualized dosing to balance efficacy, feasibility, and safety in overweight/obese populations [36, 73, 100].

#### Translational considerations and practical boundaries

Because individuals with elevated BMI often face practical and psychosocial barriers—deconditioning, joint load, and weight stigma [101] —programs should prioritize low-impact options (e.g., cycling, aquatic exercise, seated or elastic-band resistance) [102], embed behavioral support [103], and use graded progression toward WHO/ACSM activity targets [36, 104], while leveraging the acute post-exercise cognitive window identified in recent syntheses [42, 74, 105]. Even when vigorous intensities are not initially feasible, consistent moderate-intensity routines can deliver cognitive and cardiometabolic benefits; implementation should include transparent monitoring of cognitive and fitness outcomes, stepwise titration of intensity as tolerance improves, and tracking of adherence and adverse events to ensure safety and scalability [36, 73, 100].

Translation to practice requires attention to comparator conditions, timing, and task demands. Passive cognitive engagement controls may attenuate observable contrasts; thus, applications should distinguish exercise-specific benefits from nonspecific attention/expectancy effects [91–93]. Acute benefits also appear time sensitive, but evidence is insufficient to recommend a single “optimal” timing rule across tasks and populations. While higher-arousal modes may yield larger point estimates, current data do not support a universal “more intense is better” rule; a context-dependent, potentially non-linear dose–response pattern is more plausible and should be weighed against tolerability and adherence [69, 85].

Because individuals with elevated BMI may face practical and psychosocial barriers (e.g., deconditioning, joint load, and weight stigma), implementation should prioritize feasible, low-impact, progressively dosed programs with behavioral support, aligned with WHO/ACSM targets and safety monitoring [36, 73, 101–105].

Finally, certainty varied across outcomes. Because RA and N2 effects were less consistent and more imprecise, they should be treated as supportive rather than prescriptive; recommendations should prioritize more robust outcomes (RT and P3) while acknowledging residual heterogeneity and limitations in ERP reporting. Future trials that preregister task-contrast definitions, harmonize ERP pipelines, and include adequately powered training interventions will strengthen translational guidance [25, 41, 98].

### Advantages, limitations, and future directions

#### Advantages

This review focuses on overweight/obese populations and quantitatively synthesizes exercise-related changes in cognitive performance and ERP indices (P3, N2), translating findings into prescription-relevant implications. Methodologically, we applied Cochrane RoB-2 and an ERP-specific reporting checklist to anchor D4/D5 judgments and prespecified sensitivity analyses. Moderator analyses combined prespecified subgroups with REML meta-regression under Knapp–Hartung inference, and robustness was assessed via leave-one-out and small-study diagnostics (funnel/Egger, trim-and-fill where indicated), improving transparency of inferences.

#### Limitations

Despite a focused PICOS and population scope (overweight/obesity), evidence remains limited. (i) Heterogeneity was substantial (I² often ~ 70–90%) due to variability in samples, exercise protocols, control activities, and ERP pipelines; moderator analyses explained only part of this variance. (ii) Evidence depth was uneven—particularly for N2 (k = 6)—leading to imprecision and sensitivity to single studies; RA precision was also borderline. (iii) ERP D4/D5 reporting gaps (preprocessing detail, trial retention, prespecification/multiplicity control) were common, reducing certainty and comparability. (iv) Residual confounding (e.g., baseline fitness, total dose, mental health, cognitive engagement during control, washout adequacy) cannot be excluded, especially in crossover trials. (v) Generalizability is constrained by predominantly young-adult samples and limited matched comparator/timing standardization.

Although sensitivity and small-study checks were conducted (see § 3.6), their interpretability is constrained by small k in some outcomes—especially N2—so null asymmetry tests should be interpreted cautiously.

#### Future directions

Future trials should be adequately powered RCTs with preregistered outcomes and moderator hypotheses and should report within-subject correlations for crossover designs. ERP methods should be standardized and transparently reported using checklist-aligned practices addressing RoB-2 D4/D5. Comparator conditions should be clearly defined and attention-matched where feasible. Acute studies should sample ERP at ≤ 15, 16–30, and ≥ 31 min post-exercise; training studies should include baseline and end-of-program assessments (and follow-up where feasible) to separate acute from training adaptations. Exercise dose should be comprehensively characterized and, where possible, tested in factorial or multi-arm designs (mode × intensity × duration × timing). Broader sampling beyond young adults, better sex balance, and fitness/metabolic profiling, alongside adherence and adverse-event monitoring, will strengthen translation to clinical and public-health contexts.

## Conclusion

In recent years, electrophysiology has advanced rapidly—especially in recording hardware and analytic software—allowing much more precise tracking of neuroelectric dynamics across populations and experimental conditions. Work focused on people with overweight/obesity has largely emerged only recently (2019–2025), and further evidence is still needed. Our meta-analysis shows that, in randomized trials enrolling overweight/obese participants, exercise significantly shortens reaction time (RT) and increases P3 amplitude. Exercise also appears to improve response accuracy (RA) and make N2 more negative, but the certainty for these latter outcomes is lower due to heterogeneity, small sample sizes, and reporting gaps. These conclusions are consistent with mechanistic accounts of acute catecholaminergic/arousal responses (e.g., LC–NE) and training-induced vascular/neurotrophic adaptations, indicating that exercise can modulate attentional resource allocation and conflict monitoring in individuals with elevated BMI and lead to faster information processing and improved executive control.

Notably, although most prespecified moderators did not show clear between-group differences, our subgroup and meta-regression patterns suggest that exercise mode, session duration, post-exercise assessment timing, task demands, and the choice of control activity may influence the magnitude of observed effects and contribute to heterogeneity. Future studies should therefore design realistic, tailored exercise prescriptions for individuals with elevated BMI. In addition, greater standardization of ERP acquisition and analysis pipelines is needed, which will reduce measurement/selection bias (RoB-2 D4/D5), lower between-study heterogeneity, and increase certainty of evidence.

## Supplementary Information

Supplementary Material 1.

Supplementary Material 2.

Supplementary Material 3.

Supplementary Material 4.

Supplementary Material 5.

Supplementary Material 6.

Supplementary Material 7.

## Data Availability

Data relevant to this study are available from the corresponding author (ZCC) upon reasonable request.

## Abbreviations

ACSM: American College of Sports Medicine
ANCOVA: Analysis of covariance
BDNF: Brain-derived neurotrophic factor
BIDS: Brain Imaging Data Structure
BMI: Body mass index
CI: Confidence interval
EEG: Electroencephalography
ERN: Error-related negativity
ERP: Event-related potential
FDR: False discovery rate
GRADE: Grading of Recommendations Assessment, Development and Evaluation
HIIE: High-intensity interval exercise
HR: Hazard ratio
HRmax: Maximal heart rate
HRR: Heart rate reserve
I²: Heterogeneity I-squared statistic
ICA: Independent component analysis
KH: Knapp–Hartung (adjustment)
LPP: Late positive potential
MD: Mean difference
MICT: Moderate-intensity continuous training
ms: milliseconds
N2: N2 component
OR: Odds ratio
P2: P2 component
P3: P300 (P3) component
P3b: P3b subcomponent of the P300
PRISMA: Preferred Reporting Items for Systematic Reviews and Meta-Analyses
QC: Quality control
RA: Reaction accuracy
RCT: Randomized controlled trial
REML: Restricted maximum likelihood
RM: Repetition maximum
RoB: Risk of bias
RR: Risk ratio
RT: Reaction time
RPE: Rating of perceived exertion
SD: Standard deviation
SE: Standard error
SNR: Signal-to-noise ratio
SoF: Summary of Findings
VO₂max: Maximal oxygen uptake
VO₂peak: Peak oxygen uptake
µV: microvolts
ROI: Region of interest
ROIs: Regions of interest
