# Supplementary material for: Effects of exercise on cognitive performance and ERP (P3, N2) in overweight and obese populations: a systematic review and meta-analysis
Source: BMC Sports Sci Med Rehabil. 2026 Feb 16;18:151. doi: 10.1186/s13102-026-01593-0 (PMC13011643; doi:10.1186/s13102-026-01593-0)
Supplement: Supplementary file 1 — Supplementary Material 1. [file 13102_2026_1593_MOESM1_ESM.docx]

**Supplement 1: Complete Search Query**

Pubmed:

((((((exercise[MeSH Terms]) OR (exercise*[Title/Abstract])) OR (aerobic exercise[MeSH Terms])) OR (exercise, physical[MeSH Terms])) OR (physical exercise[MeSH Terms]) OR (physical activity[MeSH Terms]) OR (training, Exercise[MeSH Terms])) AND (((((((((event-related potentials[MeSH Terms]) OR (event-related potential*[Title/Abstract])) OR (Event Related Potential[MeSH Terms])) OR (Event Related Potential*[Title/Abstract])) OR (Evoked Potential*[MeSH Terms])) OR (Evoked Potential*[Title/Abstract])) OR (ERP[Title/Abstract])) OR (ERPs[Title/Abstract])))) NOT ((animals[MeSH Terms]) NOT (humans[MeSH Terms])) Filters: English, French

N=1350

Embase:

('exercise'/exp OR exercise OR exercise* OR (physical AND ('activity'/exp OR activity))) AND ('event related' AND potentials OR (evoked AND potentials) OR 'erp'/exp OR erp) AND ([english]/lim OR [french]/lim)

N=2186

Cochrane Library:

ID Search Hits

#1 MeSH descriptor: [Exercise] explode all trees 39777

#2 (exercise):ti,ab,kw (Word variations have been searched) 153327

#3 (Physical Activity):ti,ab,kw (Word variations have been searched) 81973

#4 #1 OR #2 OR #3 201662

#5 MeSH descriptor: [Evoked Potentials] explode all trees 4271

#6 (event-related potentials):ti,ab,kw (Word variations have been searched) 3080

#7 (Evoked Potentials):ti,ab,kw (Word variations have been searched) 7616

#8 (ERP):ti,ab,kw (Word variations have been searched) 2093

#9 #5 OR #6 OR #7 OR #8 10098

#10 #4 AND #9 N=961

Web of Science:

1: (((((((TS=(exercise)) OR TS=(Physical Exercise)) OR TS=(Physical Activity)) OR TS=(Training)) OR TI=(exercise*)) OR TI=(Physical Exercise)) OR TI=(Physical Activity)) OR TI=(Training) and Preprint Citation Index N=6518557

2: ((((((TS=(event-related potentials)) OR TS=(Event Related Potential)) OR TS=(Evoked Potentials)) OR TI=(event-related potential*)) OR TI=(Event Related Potential*)) OR TI=(Evoked Potential*)) OR TI=(ERP*) and Preprint Citation Index N=368167

3: (((((TS=(obese)) OR TS=(obesity)) OR TS=(overweight)) OR TI=(obese)) OR TI=(obesity)) OR TI=(overweight) and Preprint Citation Index N=1154441

4: #1 AND #2 AND #3 and Preprint Citation Index N=805

**TOTALL: N=1350+2186+954+805=5295**
